# Using clustering of genetic variants in Mendelian randomization to interrogate the causal pathways underlying multimorbidity from a common risk factor

**DOI:** 10.1002/gepi.22582

**Published:** 2024-08-13

**Authors:** Xiaoran Liang, Ninon Mounier, Nicolas Apfel, Sara Khalid, Timothy M. Frayling, Jack Bowden

**Affiliations:** ^1^ Department of Clinical and Biomedical Sciences, Faculty of Health and Life Sciences University of Exeter Exeter UK; ^2^ Department of Economics University of Southampton Southampton UK; ^3^ Centre for Statistics in Medicine, Nuffield Department of Orthopaedics, Rheumatology and Musculoskeletal Sciences University of Oxford Oxford UK; ^4^ Department of Genetic Medicine and Development, Faculty of Medicine CMU Geneva Switzerland

**Keywords:** clustering analysis, heterogeneous causal effects, hierarchical clustering, Mendelian randomization, multimorbidity, robust MR

## Abstract

Mendelian randomization (MR) is an epidemiological approach that utilizes genetic variants as instrumental variables to estimate the causal effect of an exposure on a health outcome. This paper investigates an MR scenario in which genetic variants aggregate into clusters that identify heterogeneous causal effects. Such variant clusters are likely to emerge if they affect the exposure and outcome via distinct biological pathways. In the multi‐outcome MR framework, where a shared exposure causally impacts several disease outcomes simultaneously, these variant clusters can provide insights into the common disease‐causing mechanisms underpinning the co‐occurrence of multiple long‐term conditions, a phenomenon known as multimorbidity. To identify such variant clusters, we adapt the general method of agglomerative hierarchical clustering to multi‐sample summary‐data MR setup, enabling cluster detection based on variant‐specific ratio estimates. Particularly, we tailor the method for multi‐outcome MR to aid in elucidating the causal pathways through which a common risk factor contributes to multiple morbidities. We show in simulations that our “MR‐AHC” method detects clusters with high accuracy, outperforming the existing methods. We apply the method to investigate the causal effects of high body fat percentage on type 2 diabetes and osteoarthritis, uncovering interconnected cellular processes underlying this multimorbid disease pair.

## 1 INTRODUCTION

Mendelian randomization (MR) is a widely used method in epidemiology that leverages genetic variants (usually in the form of single‐nucleotide polymorphisms [SNPs]) as instrumental variables (IV) for estimating the causal effect of a potentially confounded exposure on an outcome (Lawlor et al., 2008; Smith & Ebrahim, 2004). If a genetic variant is sufficiently associated with the exposure, independent of possible confounders of the exposure‐outcome relationship, and affects the outcome only through the exposure, then it is a valid instrument for assessing causality (Didelez & Sheehan, 2007). With further parametric assumptions, for example that relationships between all variables are additive and linear, and all variants included as instruments encode a single homogeneous causal effect from the exposure to the outcome, the causal parameter of interest can be estimated using simple meta‐analytic methods based on genome‐wide summary statistics (Bowden & Holmes, 2019; Palmer et al., 2012; Sleiman & Grant, 2010). In this setting, all the variant‐specific causal estimates are expected to target the same, true causal effect, and their “ratio” estimates (derived as the ratio of the variant‐outcome to variant‐exposure association), from which the overall meta‐analysis is performed, should vary by sampling error alone (Greco et al., 2015; Hernán & Robins, 2006). Excess “heterogeneity” amongst the ratio estimates is therefore a sign that one or more of the assumptions has been violated (Burgess et al., 2019).

A major source of excess heterogeneity is undoubtedly *horizontal pleiotropy*, the phenomenon whereby a variant affects multiple traits and therefore is associated with the outcome through pathways other than via the exposure (Burgess et al., 2019; Hemani et al., 2018). This has been extensively studied with improved methods for pleiotropy detection (Bowden et al., 2018; Greco et al., 2015) and robust estimation (Bowden et al., 2017; Bowden & Holmes, 2019; Zhao et al., 2020). Violation of the causal effect homogeneity assumption, has, by contrast, been far less researched, despite this being a plausible feature of many analyses. For example, it is suspected that general adiposity, which is often proxied by a single trait like body mass index (BMI), exerts a heterogeneous causal effect on type 2 diabetes (T2D) depending on the location of the adipose tissue in the body (e.g., if it is peripheral or visceral) (Loos & Kilpeläinen, 2018). In this case, variants associated with different physiological aspects of the exposure may target distinct causal effects.

In the presence of excess heterogeneity from both sources, the genetic variants can be grouped into distinct clusters, such that all variants in each cluster indicate the same effect. Several studies have explored variant clusters in the MR framework. It is well recognized that it is impossible to discern whether each cluster embodies genuine causal mechanisms, or is formed due to pleiotropic pathways, without further domain knowledge or modeling assumptions. Therefore, overdispersion caused by both sources can be summarized under an umbrella term such as “clustered heterogeneity,” as proposed by Foley et al. (2021) or “mechanistic heterogeneity” by Iong et al. (2024).

In this paper, we propose a method to identify variant clusters under mechanistic heterogeneity, building upon the agglomerative hierarchical clustering (AHC) method developed by Apfel and Liang (2024) in the field of econometrics for IV selection with one‐sample individual‐level data. We adapt the method to the multi‐sample summary‐data MR setting, hence referring to it as “MR‐AHC,” to group variants based on their ratio estimates using summary statistics measured from genome‐wide association studies (GWAS). Specifically, we adjust the algorithm to account for the uncertainty of the clustering objects (i.e., the ratio estimates) rather than treating them as fixed data points as in AHC. In addition, we propose an extension of the algorithm to deal with outliers amongst the ratio estimates. We showcase that our modified MR‐AHC algorithm improves the clustering accuracy over the original AHC approach, therefore representing a meaningful extension for summary‐data MR.

More notably, we have tailored the method to the multi‐outcome MR setting, in which a shared exposure causally impacts several disease outcomes simultaneously. This extension is specially crafted for investigating the common disease‐causing pathways underpinning multimorbidity, which refers to the coexistence of two or more long‐term conditions in one individual (Marengoni et al., 2011). A significant proportion of the adult population is affected by multimorbidity (Barnes, 2015). A meta‐analysis conducted by Chowdhury et al. (2023) has estimated that the global prevalence of multimorbidity is approximately 37%, with rates exceeding 50% among adults aged over 60 years. Furthermore, the forthcoming decades are expected to witness a further substantial increase in multimorbidity prevalence worldwide (Langenberg et al., 2023). Given the significant impact of multimorbidity on both individuals and society, it has been recognized as a global priority for health research (Masoli et al., 2022; Skou et al., 2022). Considerable research efforts are required to address the challenges associated with effective interventions for multimorbidity, with a key focus on exploring the common pathogenetic mechanisms linking multiple morbidities (Barnes, 2015).

For this purpose, numerous studies have identified common causal exposures underlying a broad range of conditions. New statistical approaches have also been introduced for comprehending disease causality in multimorbidity. For example, the MR^2^ method (Zuber et al., 2023) identifies shared exposures for a given set of multimorbid conditions. However, such common risk factors are often complex traits, and may exert heterogeneous influences on diseases through multifaceted mechanisms. For instance, obesity, one of the most well‐established risk factors contributing to various forms of multimorbidity (Agborsangaya et al., 2013; Skou et al., 2022), is recognized to impact diseases through a variety of distinct pathways (Loos & Kilpeläinen, 2018; Martin et al., 2022).

**Figure 1:**
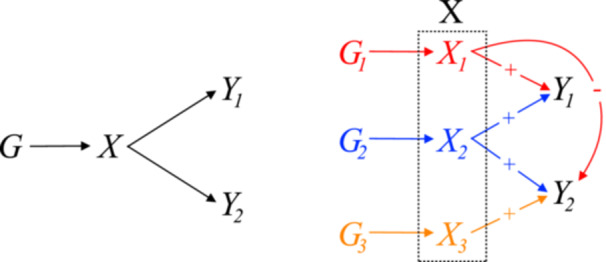
Left: Multi‐outcome MR involving two disease outcomes and a common risk factor. Right: Clusters formed by the variants associated with the common exposure, which reflect heterogeneous causal pathways.

Given the multitude of the potential causal pathways stemming from a common risk factor, especially in the case of complex traits like obesity, to enable effective clinical prevention and treatment, further investigation is necessary to elucidate the pathways through which this common risk factor induces the co‐occurrence of multiple morbidities. A starting point can be identifying the variant clusters associated with distinct causal effects within a multi‐outcome MR framework. To illustrate this, consider the hypothetical example depicted in Figure 1. Here, variants linked to the exposure X are divided into three groups (*G*_1_–*G*_3_), as they influence the two disease outcomes $Y_{1}$ and $Y_{2}$ through three different aspects of the exposure X (denoted by *X*_1_–*X*_3_) that might not be easy to measure directly. Among the three groups, $G_{1}$ is associated with an increasing effect on $Y_{1}$ but a protective effect against $Y_{2}$, and $G_{3}$ indicates an increasing effect on $Y_{2}$ but no effect on $Y_{1}$. Only the group $G_{2}$ corresponds to pathways through which the common risk factor increases the risk of both diseases. Therefore, identifying variant clusters can uncover previously hidden heterogeneous causal effects, and subsequently shed light on the shared biological pathways linking the common risk factor to the multimorbid conditions. This can be crucial for identifying the therapeutic targets for multimorbidity.

Several clustering approaches have been proposed within the MR framework to group genetic variants based on their causal estimates, such as MR‐Clust (Foley et al., 2021) and MR‐PATH (Iong et al., 2024). However, these methods are primarily tailored to settings involving a single exposure and a single outcome, making them less suitable for handling the complexities of multimorbidity, as they lack multidimensional clustering options. On the other hand, methods such as NAvMix (Grant et al., 2022) do allow for multidimensional clustering of genetic variants, but are not inherently rooted in the MR framework, since they group variants based on their direct variant‐trait associations, rather than causal estimates. This may limit their utility for causal inference. The mclust method (Scrucca et al., 2016) does permit multidimensional clustering using causal estimates, but we show that the method's accuracy can be suboptimal. In contrast, our MR‐AHC method allows for multidimensional causal clustering based on MR estimates, whilst achieving a high clustering accuracy, which we have demonstrated in extensive Monte Carlo simulations.

We apply MR‐AHC to investigate the causal effects of body fat percentage (BFP), as a shared risk factor, on a pair of multimorbid conditions, T2D and osteoarthritis (OA). Our analysis identifies four variant clusters indicating heterogeneous effects. To provide insights into the underlying causal pathways, we conducted comprehensive pathway analyses on the variant clusters, uncovering interconnected cellular processes related to gene expression transcription and cellular responses to stimuli underlying the T2D‐OA multimorbidity. We provide further evidence using cluster‐specific MR for the shared pathway through oxidative stress (OS). For the cluster exhibits a protective effect against T2D, the results suggest a possible mechanism involving enhanced activity of the ion channels related to insulin secretion. While the clustering results cannot directly label a cluster as signifying genuine causal mechanisms or pleiotropic pathways, we show how post‐clustering analyses may enable this distinction.

## 2 METHOD

### 2.1 Model setup

We assume a linear MR model allowing for multiple outcomes with a common exposure, which also accommodates horizontal pleiotropy and heterogeneous causal effects. We also assume that the causal effects from the exposure to the outcomes via different pathways are additive. Therefore, without loss of generality, we model the causality heterogeneity using additive subcomponents of the exposure. Let the common exposure X be denoted by $X=X_{1}+\cdots +X_{K}$ where $X_{k}$ is the kth unobserved subcomponent in X with k=1,…,K. Let the pth outcome be denoted by the scalar $Y_{p}$ where p=1,…,P and P≥2 in multi‐outcome MR. The vector $G=\left(G_{1},\ldots ,G_{J}\right)\prime$ is used to denote the J genetic variants used as IV. We then have the following linear structural model:

(1) $$U=\sum_{j=1}^{J}\eta_{j}G_{j}+\epsilon_{U},$$

(2) $$X_{k}=\sum_{j=1}^{J}\delta_{kj}G_{j}+q_{xk}U+\epsilon_{Xk},$$

(3) $$X=\sum_{k=1}^{K}X_{k},$$

(4) $$Y_{p}=\sum_{k=1}^{K}\theta_{kp}X_{k}+\sum_{j=1}^{J}\psi_{jp}G_{j}+q_{yp}U+\epsilon_{Yp},$$

where U represents all uncontrolled confounding between any pair of subexposures $X_{k}$ and outcome $Y_{p}$, or indeed any outcome pair. More complex confounding structures could exist between each subexposure and outcome, and are highly plausible, but they would not materially affect the execution of our method. Hence, for simplicity, we assume a common U. $\epsilon_{U},\epsilon_{Xk}$, and $\epsilon_{Yp}$ are error terms affecting $U,X_{k}$, and $Y_{p}$, respectively, and we assume $E\left(\epsilon_{U}G_{j}\right)=E\left(\epsilon_{Xk}G_{j}\right)=0$ for all j=1,…J; $\theta_{1p},\ldots ,\theta_{Kp}$ represent the heterogeneous direct causal effects of X on $Y_{p}$. Genetic variants can exert direct pleiotropic effects on the outcomes as captured by $\psi_{jp}$, and/or affect the exposure and outcomes through the common confounder *U* as the correlated pleiotropy (Morrison et al., 2020). The unmeasured phenotypic correlations between two outcomes $Y_{p}$ and $Y_{q}$ (p≠q) are summarized in the common confounding U and the possible correlations between $\epsilon_{Yp}$ and $\epsilon_{Yq}$. We show in subsequent sections how our method handle the phenotypic correlations when the outcome GWAS studies are conducted in overlapping samples.

We maintain the assumption that all the genetic variants are themselves mutually uncorrelated (i.e., not in linkage disequilibrium [LD]), and now inspect the relationship between each individual variant $G_{j}$ and X as well as between $G_{j}$ and $Y_{p}$. First, from (1) and (2), we obtain the reduced‐form relationship between $X_{k}$ and $G_{j}$ as

$$X_{k}=\gamma_{kj}G_{j}+\xi_{Xkj},$$

where the total effect of $G_{j}$ on $X_{k}$ is $\gamma_{kj}=\delta_{kj}+q_{xk}\eta_{j}$. The error term $\xi_{Xkj}$ is defined implicitly, depending on $\epsilon_{Xk},\epsilon_{U}$ and all $G_{i}$ with i≠j. From the the previous assumptions that $E\left(\epsilon_{U}G_{j}\right)=E\left(\epsilon_{Xk}G_{j}\right)=0$ and that $G_{j}$ is uncorrelated with all other variants, we have $E\left(\xi_{Xkj}G_{j}\right)=0$. It follows from (3) that the overall relationship between $G_{j}$ and the exposure X is

(5) $$X=\gamma_{j}G_{j}+\xi_{Xj},$$

where $\gamma_{j}=\sum_{k=1}^{K}\gamma_{kj}=\sum_{k=1}^{K}\delta_{kj}+\eta_{j}\sum_{k=1}^{K}q_{xk}$ and the error term $\xi_{Xj}=\sum_{k=1}^{K}\xi_{Xkj}$ with $E\left(\xi_{Xj}G_{j}\right)=0$. We assume that the relevance condition for the instruments is satisfied at the scale of the overall exposure X so that $\gamma_{j}\neq 0$ for j=1,…,J.

Now we inspect the reduced‐form relationship between $G_{j}$ and $Y_{p}$. It follows from (1) and (4) that the pleiotropic effect of $G_{j}$ on $Y_{p}$ can be derived as $\alpha_{jp}=\psi_{jp}+q_{yp}\eta_{j}$. In addition, by plugging (2) into (4), the overall reduced‐form between $Y_{p}$ and $G_{j}$ is

(6) $$Y_{p}=\Gamma_{jp}G_{j}+\xi_{Ypj},$$

where $\Gamma_{jp}=\sum_{k=1}^{K}\theta_{kp}\gamma_{kj}+\alpha_{jp}=\sum_{k=1}^{K}\theta_{kp}\delta_{kj}+\eta_{j}\left(\sum_{k=1}^{K}\theta_{kp}q_{xk}+q_{yp}\right)+\psi_{jp}$. The correlation between the implicitly‐defined error term $\xi_{Ypj}$ and $G_{j}$ depends on the correlation between $\epsilon_{Yp}$ from Equation (4) and $G_{j}$. If $E\left(\epsilon_{Yp}G_{j}\right)=0$, then $\xi_{Ypj}$ and $G_{j}$ are also uncorrelated. In this case, for the $G_{j}$‐$Y_{p}$ association estimate (denoted by ${\hat{\Gamma }}_{jp}$) generated from a GWAS by regressing $Y_{p}$ on $G_{j}$ in a given sample, we have

$${\hat{\Gamma }}_{jp}\overset{p}{\to }\Gamma_{jp}$$

as the sample size n→∞. Similarly, for the $G_{j}$‐X association estimated in a GWAS (denoted by ${\hat{\gamma }}_{j}$) in a sample independent from the $G_{j}$‐$Y_{p}$ sample, we have

$${\hat{\gamma }}_{j}\overset{p}{\to }\gamma_{j}.$$

Then for the variant‐specific causal estimate of $G_{j}$, defined as ${\hat{\beta }}_{jp}={\hat{\Gamma }}_{jp}∕{\hat{\gamma }}_{j}$, we have

$${\hat{\beta }}_{jp}\overset{p}{\to }\beta_{jp}\;\;\text{and}\;\;\beta_{jp}=\frac{\Gamma_{jp}}{\gamma_{j}}=\frac{\sum_{k=1}^{K}\theta_{kp}\gamma_{kj}+\alpha_{jp}}{\sum_{k=1}^{K}\gamma_{kj}}.$$

In words, as the sample size n goes to infinity, each ${\hat{\beta }}_{jp}$ converges to their variant‐specific causal estimand $\beta_{jp}$, which is the causal effect from X to $Y_{p}$ identified using only $G_{j}$ as instrument. In the simple case where $G_{j}$ only instruments one subcomponent $X_{k}$, the variant‐specific causal estimand then becomes:

(7) $$\beta_{jp}=\frac{\theta_{kp}\gamma_{kj}+\alpha_{jp}}{\gamma_{kj}}=\theta_{kp}+\frac{\alpha_{jp}}{\gamma_{kj}}.$$

Equation (7) reflects the possible sources of mechanistic heterogeneity among the variant‐specific estimates: heterogeneous causal effect from the exposure to the outcome, and pleiotropic effects. We aim to group the genetic variants into distinct clusters such that within each cluster, all variants identify the same effect. More generally, for the multi‐outcome MR with P≥2, for a given variant $G_{j}$, we combine all ${\hat{\beta }}_{jp}$ and $\beta_{jp}$ for p=1,…,P into the vectors ${\hat{\beta }}_{j}=\left({\hat{\beta }}_{j1},\ldots ,{\hat{\beta }}_{jP}\right)\prime$ and $\beta_{j}=\left(\beta_{j1},\ldots ,\beta_{jP}\right)\prime$. We propose the MR‐AHC method, elaborated in the subsequent sections, to divide the genetic variants into distinct clusters based on the similarity of their ratio estimates ${\hat{\beta }}_{j}$, so that variants identifying the same estimand $\beta_{j}$ are assigned into the same cluster. We provide further discussion on mechanistic heterogeneity under different parameter specifications in Appendix SC.1.

Thus far, we have inspected the case where there is no residual correlation between $G_{j}$ and $\epsilon_{Yp}$ in Equation (4), that is, $E\left(\epsilon_{Yp}G_{j}\right)=0$. In a multi‐outcome MR model, this can be violated if there is direct causality between the outcome variables. For example, consider two outcomes $Y_{p}$ and $Y_{q}$ (p≠q), if $Y_{q}$ causally affects $Y_{p}$ directly, then it will enter Equation (4) as part of $\epsilon_{Yp}$, hence the error term may be correlated with $G_{j}$. We show in Appendix SC.2 that the clustering results of the variants are in general not affected by the additional direct causality between the outcomes, but the causal effects identified by each cluster are the total effects including the outcomes causality, instead of the direct effects from the exposure to the outcomes. In this paper, we mainly focus on the case without the direct outcome causality.

### 2.2 The MR‐AHC method for clustering genetic variants in summary‐data Mendelian randomization

Within the aforementioned MR framework, we propose the MR‐AHC method for clustering genetic variants using the following summary statistics measured from GWAS for J uncorrelated genetic variants: the variant‐exposure association estimate ${\hat{\gamma }}_{j}$, and the variant‐outcome association estimate ${\hat{\Gamma }}_{j}$, where j=1,…,J. In an MR setting with a common exposure and multiple P outcomes, ${\hat{\Gamma }}_{j}$ is the vector of the P variant‐outcome associations ${\hat{\Gamma }}_{jp}$ with p=1,…,P. The causal estimates of the exposure on the P outcomes using only variant j as instrument (i.e. the ratio estimates) can then be obtained as the P‐dimensional vector ${\hat{\beta }}_{j}={\hat{\Gamma }}_{j}∕{\hat{\gamma }}_{j}$. We maintain the assumption that the variant‐exposure associations are measured from a sample independent from all the variant‐outcome association samples, but overlap between the outcome samples is allowed.

We make the following normality assumption on the summary statistics:

$$\sqrt{n}\left(\left(\begin{array}{l} {\hat{\Gamma }}_{jp}\\ {\hat{\gamma }}_{j} \end{array}\right)-\left(\begin{array}{l} \Gamma_{jp}\\ \gamma_{j} \end{array}\right)\right)\overset{d}{\to }N\left(\left(\begin{array}{l} 0\\ 0 \end{array}\right),\left(\begin{array}{ll} \sigma_{Yjp}^{2} & 0\\ 0 & \sigma_{Xj}^{2} \end{array}\right)\right),$$

for j=1,…,J and p=1,…,P. The estimates of the standard errors of ${\hat{\gamma }}_{j}$ and ${\hat{\Gamma }}_{jp}$, denoted by $se\left({\hat{\gamma }}_{j}\right)$ and $se\left({\hat{\Gamma }}_{jp}\right)$, are generally given by the corresponding GWAS, hence taken as known. It follows that $\sqrt{n}\left({\hat{\beta }}_{j}-\beta_{j}\right)\overset{d}{\to }N(0,{𝚺}_{j}^{A})$ where $\beta_{j}=\Gamma_{j}∕\gamma_{j}$ are the causal effects identified by the jth variant and ${𝚺}_{j}^{A}$ is the P×P asymptotic covariance matrix. Let ${\hat{\Sigma }}_{j}$ be an estimate of the covariance matrix of ${\hat{\beta }}_{j}$. The diagonal entries of ${\hat{\Sigma }}_{j}$ are the variance estimates of ${\hat{\beta }}_{j}$, which can be typically approximately as $Var\left({\hat{\beta }}_{jp}\right)=se{\left({\hat{\Gamma }}_{jp}\right)}^{2}∕{\hat{\gamma }}_{j}^{2}$ for p=1,…,P.

**Figure 2:**
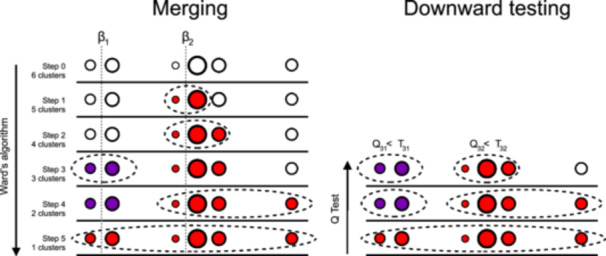
Illustration of the MR‐AHC method for a hypothetical example adapted from Apfel and Liang (2024). Left panel: Ward's algorithm defines a clustering path. Right panel: A downward testing procedure is applied until the step‐specific heterogeneity statistics cannot be rejected at a specified threshold.

Variants form a cluster if they indicate the same causal effects $\beta_{j}$, or in other words, have similar observed ratio estimates ${\hat{\beta }}_{j}$. MR‐AHC is a two‐step procedure using ${\left\{{\hat{\beta }}_{j},{\hat{\Sigma }}_{j}\right\}}_{j=1}^{J}$ as inputs to detect the variant clusters. We illustrate the method with a hypothetical example shown in Figure 2. For ease of illustration, we consider the case with a single outcome, but the same procedure applies generally with multiple outcomes. The first step of the method, the merging step, is illustrated in the left panel of Figure 2. It shows a situation with six variants that form three clusters (one of them comprised of a single variant). The dotted lines at $\beta_{1}$ and $\beta_{2}$ are the true heterogeneous causal effects, and the circles above the real line denote the variant‐specific ratio estimates. The differences in the size of the circles reflect the fact that summary‐data estimates exhibit varying degrees of uncertainty. In the explanation below, we refer to these estimates and their corresponding variants by the numbers 1–6, from left to right.

In the initialization step of the merging process, each variant‐specific estimate forms its own cluster. Next, we merge the two estimates which are closest in terms of their weighted squared Euclidean distance, that is, those estimated with Variant 3 and 4. These two estimates are merged into one cluster and we now have five clusters left. We recalculate the pairwise distances with the five clusters and merge the closest two into a new cluster. Continue with this procedure until Step 5 where all variants are in a single cluster.

By the end of the merging step, we have generated a clustering path. Along each step of the path, the number of clusters, denoted by K, varies from K=1 to K=J by increments of 1. Next, in the second step of MR‐AHC, we retrace the clustering path to select the optimal value of K using a downward testing procedure, operates as follows: starting from the largest cluster containing all variants, apply Cochran's *Q* test (Cochran, 1950) to examine the degree of heterogeneity of all the ratio estimates by calculating the test statistic and comparing it with a prespecified significance threshold. If the null hypothesis of “no excess heterogeneity” gets rejected, then move to the next level of the clustering path and apply the *Q* test to all the subclusters on that level. Repeat this process until reaching a level where no subcluster heterogeneity statistic rejects at the given significance threshold. In our illustrative example, we would expect the downward testing procedure to retrace from Step 5 to Step 3 of the clustering path, thus determining three clusters formed by Variants 1–2, Variants 3–5, and Variant 6 alone.

In the original AHC algorithm proposed by Apfel and Liang (2024), the inputs are essentially just the ratio estimates ${\hat{\beta }}_{j}$, hence the clustering objects are treated as nonrandom fixed data points. MR‐AHC adjusts the algorithm to take into account the uncertainty of ${\hat{\beta }}_{j}$ by incorporating the covariance matrix ${\hat{\Sigma }}_{j}$ into the weighted squared Euclidean distance in the merging process. We show in Appendix SA that this distance is essentially the Wald statistic for testing the null hypothesis that “the two clusters indicate the same causal effect.” Therefore, merging two clusters with the smallest distance can be interpreted as merging two clusters with the highest similarity in their cluster‐specific causal effects. To further improve the clustering accuracy, we extend the basic algorithm illustrated above to an outlier‐robust version, to correct for the outliers in the ratio estimates (see Section 2.3 for details). We illustrate the methodological improvement of MR‐AHC over AHC in simulations (See Appendix SD.3).

For the covariance matrix estimate ${\hat{\Sigma }}_{j}$, we show in Appendix SA that if all the outcome samples are nonoverlapping and/or the phenotypic correlations between the outcome traits are zero, then all the ratio estimates of a given variant are uncorrelated. In this case, all the covariance terms are zero and ${\hat{\Sigma }}_{j}$ is just a diagonal matrix with the non‐zero entries being the variances of the ratio estimates, which can be easily estimated from the GWAS summary statistics. If the covariances are non‐zero, we show in Appendix SA that ${\hat{\Sigma }}_{j}$ can be estimated via LD score regression (Bulik‐Sullivan et al., 2015). Nevertheless, we demonstrate in simulations that it is in general not a significant concern even if the covariances are treated as zero.

In the downward testing procedure, following the recommendation in Belloni et al. (2012), we define the threshold *p* value for the *Q* test as ζ=0.1/log(n) where n is the sample size. We prove in Appendix SB that this threshold *p* value results in a consistent clustering procedure. That is, as n increases, the probability of correctly identifying all true members of each cluster tends to 1. If the exposure and outcome samples are of different sizes, we recommend using the sample size of the smallest outcome sample. For binary outcomes, an effective sample size can be approximated with the number of cases and controls, see Han and Eskin (2011).

MR‐AHC does not require prespecification of the number of clusters. It can also easily identify a “null cluster” and a “junk cluster,” following the terminology of Foley et al. (2021), which refer to, respectively, the cluster identifying a zero causal effect, and the cluster containing variants not assigned to any detected clusters. Specifically, we conduct a post‐clustering Wald test on each cluster‐specific causal estimate for the null hypothesis of a zero causal effect using ζ=0.1/log(n) as the threshold *p* value. For the junk cluster, we simply classify all variants that do not fit into any other clusters as junk variants.

### 2.3 A formal description of the MR‐AHC algorithm

With ${\left\{{\hat{\beta }}_{j},{\hat{\Sigma }}_{j}\right\}}_{j=1}^{J}$ as inputs, we apply MR‐AHC in two steps: Step 1 (agglomerative hierarchical clustering) generates a decision path from K=J to K=1 clusters; Step 2 (downward testing) re‐traces the path from K=1 to K=J until the optimal cluster choice $K_{opt}$ is chosen. The first step is summarized as follows:

Step 1 Ward's algorithm (Ward, 1963)

1. **Initialization:** Each variant‐specific estimate is viewed as a cluster on its own. Hence, initially, the total number of clusters is K=J.
2. **Merging:** The two clusters that are closest as measured by their weighted squared Euclidean distance are merged into a new cluster. Without loss of generality, assume this is satisfied by cluster $S_{k}$ and $S_{l}.{\hat{\beta }}_{S_{k}}^{IVW}$ is defined as the inverse‐variance weighted (IVW) (Bowden & Holmes, 2019) mean of all the variant‐specific estimates in $S_{k}$, as follows:
$${\hat{\beta }}_{S_{k}}^{IVW}={\left({\hat{\beta }}_{S_{k},1}^{IVW},\ldots ,{\hat{\beta }}_{S_{k},P}^{IVW}\right)}^{\prime }$$
  where
(8) $${\hat{\beta }}_{S_{k},p}^{IVW}=\frac{\sum_{j\in S_{k}}{\hat{\beta }}_{jp}w_{jp}}{\sum_{j\in S_{k}}w_{jp}}$$
  with $w_{jp}=1∕v_{jp}^{2}$ for $p=1,\ldots ,P.{\hat{\beta }}_{S_{l}}^{IVW}$ for Cluster $S_{l}$ can be defined similarly. Then the weighted squared Euclidean distance between $S_{k}$ and $S_{l}$ is defined as
(9) $$D_{k,l}={\left({\hat{\beta }}_{S_{k}}^{IVW}-{\hat{\beta }}_{S_{l}}^{IVW}\right)}^{\prime }{\hat{\Omega }}_{k,l}^{-1}\left({\hat{\beta }}_{S_{k}}^{IVW}-{\hat{\beta }}_{S_{l}}^{IVW}\right).$$
  The P×P matrix ${\hat{\Omega }}_{k,l}$ is defined as follows: let $W_{kp}=\sum_{j\in S_{k}}w_{jp}$, and $W_{lp}=\sum_{j\in S_{l}}w_{jp}$ for p=1,…,P. Consider the entry at the ith column and the rth row of ${\hat{\Omega }}_{k,l}$ with i,r∈{1,…,P}, denoted by $cov_{ir}$. We have
$$cov_{ir}=\frac{\rho_{ir}}{W_{ki}W_{kr}}\sum_{j\in S_{k}}\frac{{\hat{\gamma }}_{j}^{2}}{se\left({\hat{\Gamma }}_{ji}\right)se\left({\hat{\Gamma }}_{jr}\right)}+\frac{\rho_{ir}}{W_{li}W_{lr}}\sum_{j\in S_{l}}\frac{{\hat{\gamma }}_{j}^{2}}{se\left({\hat{\Gamma }}_{ji}\right)se\left({\hat{\Gamma }}_{jr}\right)},$$
  where $\rho_{ir}$ is the correlation between ${\hat{\Gamma }}_{ji}$ and ${\hat{\Gamma }}_{jr}$, which is assumed to be constant across j=1,…,J. See Appendix SA for details.
3. **Iteration:** The merging step is repeated until all the variant‐specific estimates are in one cluster of size J.

After generating the clustering path using Step 1, we are left with a K=1 super‐cluster containing all variants. We then retrace the pathway to select the optimal value of K using a downward testing procedure originally proposed by Andrews (1999), operating as follows:

Step 2 Downward testing procedure.

Firstly, define $Q_{fg}$ to be the Cochran's Q statistic (Cochran, 1950) associated with the gth cluster at level f of the clustering path, denoted by $S_{fg}$. Also define $T_{fg}$ to be the (1−ζ) significance threshold of a $\chi^{2}$ distribution on $P\times \left(|S_{fg}|-1\right)$ degrees of freedom with ζ=0.1∕log(n), n is the sample size, and $|S_{fg}|$ is the number of variants in $S_{fg}.Q_{fg}$ is defined as follows: for the pth outcome, let $\hat{\beta^{p}}$ be the vector of length $|S_{fg}|$ with the jth entry being ${\hat{\beta }}_{jp}$ where $j\in S_{fg}$. Combine all $\hat{\beta^{p}}$ into a vector of length $P\times |S_{fg}|$ for all p=1,…,P, denoted by $B_{fg}$. Let ${\hat{\beta }}_{IVW}^{p}$ be the IVW mean of all the estimates in $\hat{\beta^{p}}$ as defined in (8), and ι be a vector of 1 of length $|S_{fg}|$. Then combine all the $|S_{fg}|$‐length vector $\iota {\hat{\beta }}_{IVW}^{p}$ into a vector of length $P\times |S_{fg}|$ for all p=1,…,P, denoted by $B_{fg}^{IVW}$. Then $Q_{fg}$ is

(10) $$Q_{fg}={\left(B_{fg}-B_{fg}^{IVW}\right)}^{\prime }{\hat{\Phi }}_{fg}^{-1}\left(B_{fg}-B_{fg}^{IVW}\right),$$

where ${\hat{\Phi }}_{fg}$ is a matrix that can be partitioned into P×P blocks. The block on the ith column and rth row, denoted by ${\hat{\Phi }}_{ir}$, is a $|S_{fg}|\times |S_{fg}|$ dimension diagonal matrix. The jth diagonal entry equals to $\frac{\rho_{ir}se\left({\hat{\Gamma }}_{ji}\right)se\left({\hat{\Gamma }}_{jr}\right)}{\gamma_{j}^{2}}$ for $j\in S_{fg}$.

1. Starting from the cluster that contains all the variants, calculate the global Q statistic, $Q_{11}$, on all the ratio estimates;
2. If $Q_{11}$ < $T_{11}$, then stop and assume that all the variants form a single cluster. If $Q_{11}\geq T_{11}$, then revert to the variant clusters on the next level of the path, where the number of clusters is K = 2;
3. Calculate Q statistics for the two subclusters separately, $Q_{21}$ and $Q_{22}$;
4. If both $Q_{21}$ < $T_{21}$ and $Q_{22}$ < $T_{22}$, then stop. Otherwise, continue to the next level where K = 3;
5. Repeat Steps 3–4 until a K∈(1,…,J) is arrived at for which no subcluster heterogeneity statistic rejects at its given threshold.

In implementing the MR‐AHC method, in addition to the baseline procedure summarized in Steps 1 and 2, we propose an extension of the method to handle outliers in the ratio estimates: after we run Steps 1 and 2 and obtain the clustering results, within each detected cluster, calculate each individual variant's contribution to the overall *Q* statistic. The individual *Q* statistic, calculated using (10) with only estimates of that variant, approximately follows a $\chi_{P}^{2}$ distribution (Bowden et al., 2018), and variants with large individual *Q* (here defined as the p‐value of the individual *Q* below 5%) are viewed as outliers. We remove the outliers from each detected cluster, and rerun Steps 1 and 2 with all the remaining variants. All the outliers are then assigned to the junk cluster.

## 3 MONTE CARLO SIMULATIONS

### 3.1 Simulation designs and methods for comparison

We evaluate the performance of MR‐AHC in various simulation settings that mimic the multimorbidity scenarios we are interested in, which involve a shared exposure causally affecting multiple outcome conditions. We consider 12 simulation designs, where the number of outcomes is either P=2 or P=3, the number of substantive variant clusters is either K=1 or K=4, and the sample correlation between the outcomes is either ρ=0,ρ=0.2, or ρ=0.7 (see Section 2.3 for the definition of ρ). In all designs, we have J=100 SNPs with 10 designated as true “junk” variants.

**Figure 3:**
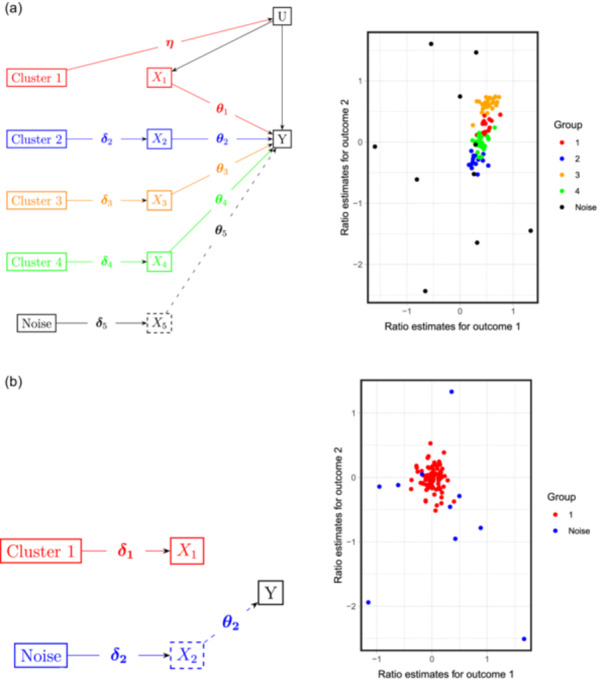
(Left in each panel) Directed acyclic graphs (DAG) of the data generation process and (right in each panel) scatter plots of representative simulated data (with two outcomes) of the simulation designs with different numbers of variant clusters. In the scatter plots, on the *x*‐axis are the ratio estimates for the first outcome, and on the *y*‐axis for the second outcome. Each point represents a specific variant. (a) Simulation designs with four substantive variant clusters. (b) Simulation designs with one substantive variant cluster.

The two classes of scenarios stratified by the number of variant clusters are illustrated in Figure 3. The directed acyclic graph (DAG) in Panel (a) illustrates the data generation process when there are four substantive clusters and one noise cluster. Multiple outcomes (two or three) are represented by the single notation Y. Variant clusters are formed due to differential sub‐components of the exposure, denoted by $X_{1}$ to $X_{5}$. Variant Cluster 1 and $X_{1}$ represent a correlated pleiotropy pathway, and Clusters 2–4 correspond to genuine heterogeneous causal mechanisms from the exposure to the outcomes. The scatter plot on the right of Panel (a) is based on a representative simulated dataset of the two‐outcome case. We also examine the performance of the method when there is actually no mechanistic heterogeneity, that is, there is only one real cluster and one noise cluster. The design is shown by a DAG and representative dataset in Figure 3b. The arrow from $X_{1}$ to Y is absent, meaning that the only substantive cluster is also a null cluster and there is no causal effect between the exposure and the outcomes.

We compare MR‐AHC with two other multidimensional clustering approaches. The first is mclust, one of the most widely used clustering method based on Gaussian mixture models (Scrucca et al., 2016). We implement mclust with ratio estimates as input in two ways: the basic setting without a noise component, and the setting incorporating a Poisson noise component. The second method is NAvMix (Grant et al., 2022) which groups genetic variants based on the variant‐trait associations instead of the ratio estimates. We choose this method as it is also motivated by interrogating the disease‐causing pathways that can possibly be revealed by genetic variants clusters. We employed two sets of inputs for NAvMix: the variant‐outcome associations, as initially proposed; and the ratio estimates as in MR‐AHC. As a general clustering method for mechanistic heterogeneity, MR‐AHC also works in the one‐outcome case. We compare MR‐AHC with MR‐Clust (Foley et al., 2021), a popular method for conducting one‐dimensional clustering based on ratio estimates, see Table S4 in Appendix SD.3. We refrain from a direct comparison with the MR^2^ method (Zuber et al., 2023), despite it also being designated for causal inference in multimorbidity. This is because MR^2^ aims for identifying shared exposures for multimorbidity and models multiple distinct exposures under strict causal effect homogeneity, while MR‐AHC looks into the potentially heterogeneous causal effects of a single shared exposure. Details of the simulation setup and implementation of the methods can be found in Appendices SD.1 and SD.2.

### 3.2 Simulation results

**Figure 4:**
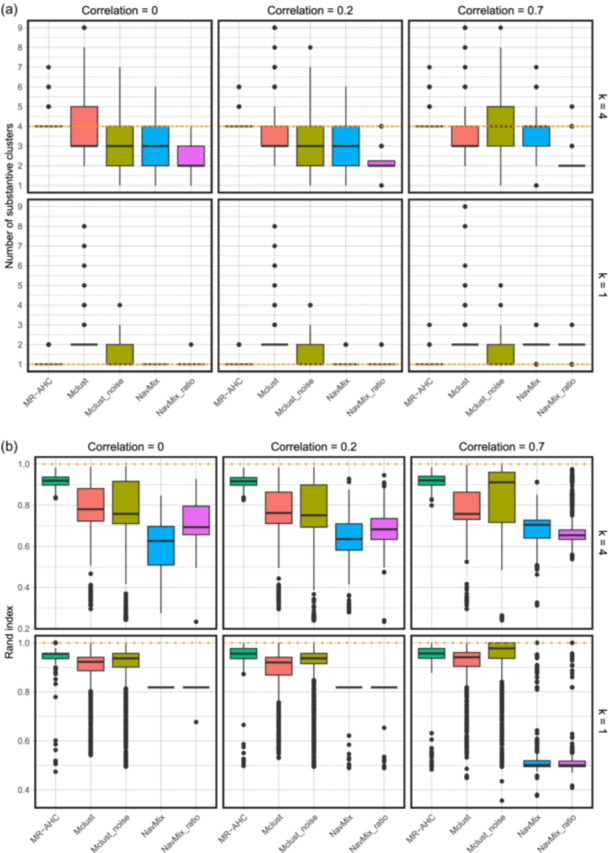
Two outcomes—boxplots for the number of detected substantive clusters and Rand index with different cluster numbers (K=4 or K=1) and outcome correlations (ρ=0,ρ=0.2, and ρ=0.7). All methods are conducted treating the outcome correlations as 0. The dotted horizontal lines represent the true values. “*mclust noise*” stands for the mclust algorithm with a noise component, and “*NAvMix ratio*” for the NAvMix method with ratio estimates as input. Results are based on 1000 replications. (a) The number of substantive clusters. (b) Rand index for variants in the substantive clusters.

For scenarios involving non‐zero outcome sample correlations, since the GWAS results typically lack a direct estimate for the correlation, we initially apply all methods treating the correlation ρ as zero. The two‐outcome simulation results are presented in Figure 4 and Table 1. Across all settings, MR‐AHC consistently demonstrates high accuracy in identifying the number of substantive clusters (“*#clusters*”), aligning closely with the ground truth in both mean and median assessments. In the boxplot of MR‐AHC (Figure 4a), all the quantiles are concentrated around the median, showing that the method consistently detects the correct number of clusters with little fluctuation. By comparison, both settings of mclust tend to underestimate the number of clusters when K=4 and overestimate it when K=1. The NAvMix method, employing two different sets of input, also exhibits a tendency to underestimate the cluster number when K=4. While it successfully identifies one cluster when K=1 with low outcome correlations, it overestimates the cluster number when the outcome correlation is high (ρ=0.7). MR‐AHC also performs very well in terms of grouping the non‐noise variants correctly, as measured by the Rand index (“*Rand index*”). It consistently achieves Rand indices close to 1, significantly outperforming all other approaches in all settings. One potential drawback of MR‐AHC is its tendency to assign slightly more noise variants to the “junk” cluster (“*#junk variants*”) than the true count, but the number of true noise variants selected as junk (“*correct junk*”) of MR‐AHC is only marginally lower than that of mclust with the noise component, outperforming all other approaches. Regarding estimation bias, both MR‐AHC and NAvMix with ratio estimates exhibit comparable mean absolute error (“*MAE*”) and mean squared error (“*MSE*”), both of which are smaller than those of other methods in most of the settings. For scenarios with K=1, MR‐AHC accurately identifies the null cluster with frequencies close to 1 (“*Freq.null*”). The two variations of NAvMix also exhibit high accuracy in this aspect, although this accuracy diminishes for NAvMix with ratio estimates when the outcome correlation is high. Definitions of the reported statistics can be found in Appendix SD.3.

**Table 1:** Simulation results for designs with two outcomes.

	MR‐AHC	mclust	mclust noise	NAvMix	NAvMix ratio
Two outcomes, *K* = 4, correlation = 0					
# clusters	4.196	3.716	2.964	3.018	2.307
Rand index	0.917	0.757	0.737	0.615	0.710
# junk variants	10.966	0.000	7.726	6.517	4.080
# correct junk	6.975	0.000	7.134	1.446	3.951
MAE	0.088	0.120	0.123	0.162	0.106
MSE	0.030	0.041	0.041	0.052	0.035
Two outcomes, *K* = 4, correlation = 0.2					
# clusters	4.207	3.665	2.933	3.130	2.254
Rand index	0.915	0.744	0.725	0.630	0.694
# junk variants	10.916	0.000	7.835	6.930	3.999
# correct junk	7.031	0.000	7.234	1.493	3.906
MAE	0.088	0.118	0.122	0.153	0.101
MSE	0.030	0.039	0.040	0.048	0.032
Two outcomes, *K* = 4, correlation = 0.7					
# clusters	4.229	3.652	3.754	3.649	2.202
Rand index	0.918	0.784	0.835	0.684	0.677
# junk variants	10.993	0.000	8.044	5.347	3.925
# correct junk	7.144	0.000	7.433	1.302	3.844
MAE	0.089	0.091	0.088	0.119	0.087
MSE	0.029	0.027	0.027	0.035	0.024
Two outcomes, *K* = 1, correlation = 0					
# clusters	1.026	2.211	1.312	1.000	1.001
Rand index	0.947	0.887	0.895	0.818	0.818
# junk variants	10.194	0.000	8.211	0.000	0.000
# correct junk	7.703	0.000	7.073	0.000	0.000
MAE	0.012	0.020	0.015	0.017	0.017
MSE	0.000	0.004	0.001	0.000	0.000
Freq.null	0.955	0.726	0.767	0.998	0.997
Two outcomes, *K* = 1, correlation = 0.2					
# clusters	1.019	2.226	1.297	1.009	1.007
Rand index	0.949	0.881	0.893	0.816	0.816
# junk variants	10.275	0.000	8.438	0.058	0.060
# correct junk	7.833	0.000	7.199	0.009	0.008
MAE	0.012	0.021	0.015	0.017	0.018
MSE	0.000	0.004	0.001	0.000	0.001
Freq.null	0.965	0.724	0.791	0.996	0.990
Two outcomes, *K* = 1, correlation = 0.7					
# clusters	1.025	2.221	1.296	1.883	1.881
Rand index	0.948	0.910	0.924	0.546	0.545
# junk variants	9.685	0.000	9.458	29.057	27.936
# correct junk	7.995	0.000	8.323	5.099	5.020
MAE	0.013	0.019	0.014	0.026	0.113
MSE	0.000	0.003	0.001	0.001	0.015
Freq.null	0.954	0.760	0.785	0.928	0.102

*Note*: All methods are conducted treating the outcome correlations as 0. Statistics are calculated as the mean over 1000 replications.

Abbreviations: mclust noise, mclust algorithm with a noise component; NAvMix ratio, NAvMix method with ratio estimates as input.

When K=4 with non‐zero outcome correlations, MR‐AHC tends to identify more clusters than the ground truth. This feature can be rectified by incorporating accurate outcome correlation information, see the results generated by applying the method with the true correlation parameter (Table S2 in Appendix SD.3). We show in Appendix SA that the outcome correlation depends on both the extent of sample overlap between the outcome samples, and the phenotypic correlation between the outcome traits. Hence, high outcome correlations are uncommon in practice. To achieve, for instance, a correlation of ρ=0.7, one may need perfect sample overlap and a phenotypic correlation of 0.7 between the two outcome traits. Even in this extreme scenario, implementing MR‐AHC while assuming a zero correlation performs reasonably well. The simulation results for scenarios with P=3 outcomes are presented in Appendix SD.3, Tables S1 and S3. Once again, MR‐AHC exhibits good performance, producing clustering results that closely align with the ground truth and generally surpassing the performance of all other approaches. In additional to the main simulations described above, we conduct extended simulations to evaluate the methods regarding post‐clustering inference as well as their performance with alternative pleiotropy parameter specification and with weak IV, see Appendix SE.

## 4 APPLICATION: ESTIMATING THE CAUSAL EFFECTS OF HIGHER ADIPOSITY ON T2D AND OA

### 4.1 Datasets and methods

We apply the MR‐AHC method to investigate the causal relationship between BFP, as a measure of adiposity, and a pair of multimorbid conditions, T2D and OA. We use a three‐sample summary‐data MR design with 487 SNPs associated with BFP as instruments, accounting for the causal effects of the common risk factor BFP on both of the conditions. The SNP‐BFP summary data are taken from a GWAS based on UK Biobank individuals from Martin et al. (2022), including 696 SNPs at genome‐wide significance ($p<5\times 10^{-8}$). The T2D GWAS statistics are from Mahajan et al. (2018), which combine 31 published GWAS studies excluding the UK Biobank individuals. The SNP‐OA summary statistics are from a FinnGen GWAS (code: M13_ARTHROSIS_INCLAVO) (FinnGen, 2022). Only SNPs present in all three datasets are used for analyses (487 in total). SNPs are orientated across all three datasets in the direction of increasing the exposure. The T2D and OA samples are nonoverlapping, therefore for each SNP, the covariance between the SNP‐T2D association estimate and SNP‐OA association estimate is treated as zero.

In implementing the MR‐AHC method, we use the effective sample size (Han & Eskin, 2011) of the T2D GWAS sample (n=193,440) to calculate the threshold *p* value 0.1/log(n) in the binary outcome setting for the Cochran's *Q* test and the post‐selection Wald test in detecting the null clusters. Clustering results of MR‐AHC are obtained using an iterated outlier removal procedure: this performs the outlier removal and re‐fitting indefinitely until the individual *p* values of the *Q* statistics for all SNPs are above 5%. The cluster‐specific causal estimates and standard errors are calculated with the IVW approach. For clusters with overdispersion indicated by a non‐zero $I^{2}$, the estimates are obtained using the robust adjusted profile score method (MR‐RAPS) (Zhao et al., 2020) to account for the within‐cluster overdispersion. For comparison, we also perform variant clustering using mclust and NAvMix (with SNP‐outcome associations as input). We set the initial proportion of noise SNPs as 5% for both methods.

### 4.2 Clustering results

**Figure 5:**
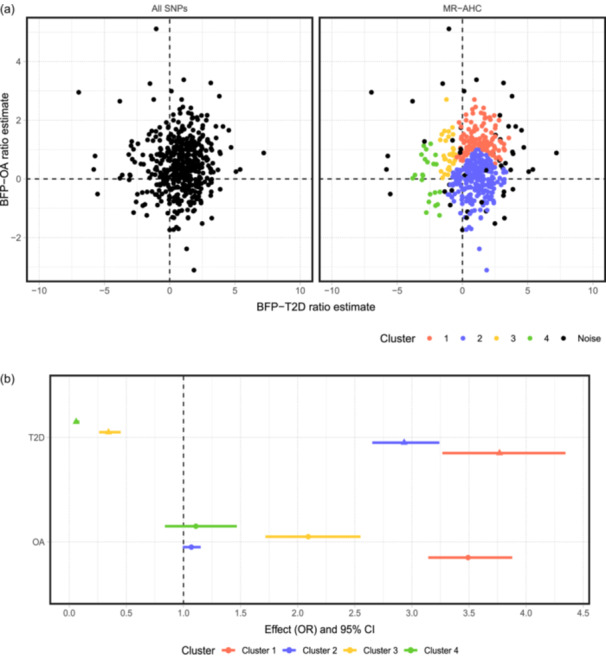
MR‐AHC clustering and estimation results of the 487 SNPs associated with body fat percentage based on their ratio estimates on type 2 diabetes and osteoarthritis. (a) (Left) The scatter plot of the 487 SNPs associated with body fat percentage; on the *x*‐axis are the ratio estimates for Type 2 diabetes, and *y*‐axis for osteoarthritis. Each point represents a specific SNP. (Right) The clustering results of MR‐AHC. (b) The cluster‐specific inverse variance weighted estimates and 95% confidence intervals in odds ratio for each cluster detected by MR‐AHC.

The clustering results of MR‐AHC are presented in Figure 5a. It detects four substantive clusters indicating heterogeneous causal effects. The cluster‐specific estimation results, obtained with the IVW approach, are depicted in Figure 5b. Among the four clusters, Cluster 1 with 124 SNPs is the only cluster associated with increasing risk for both conditions; Cluster 2 with 258 SNPs indicates an increasing risk for T2D but a null effect for OA; both Cluster 3 (32 SNPs) and Cluster 4 (22 SNPs) are associated with a protective effect against T2D, and for OA, a causative effect and a null effect, respectively. See Appendix SF.1 Table S14 for detailed estimation results.

These results align with the conclusions drawn from previous research. For example, Martin et al. (2022) examined the causal effects of higher adiposity on a variety of conditions including T2D and OA. Their findings suggest that adiposity exerts heterogeneous effects on the risk of T2D: in general, higher adiposity increases the risk of T2D, but there is a metabolically “favorable” component of adiposity that reduces the risk of the condition. For OA, all adiposity measures, including the metabolically favorable adiposity, consistently identify an increasing risk. This suggests a non‐metabolic weight‐bearing effect as a likely cause. Given this, it is reasonable to partition the variants into distinct clusters along both outcome dimensions: on the T2D‐estimate dimension, clustering occurs due to the indication of opposing effects by different variants; on the OA‐estimate dimension, clustering is also likely to occur, as we may expect an adverse effect if the variants are associated with fat located around the articulations in a load‐bearing way, but no effect elsewhere.

**Figure 6:**
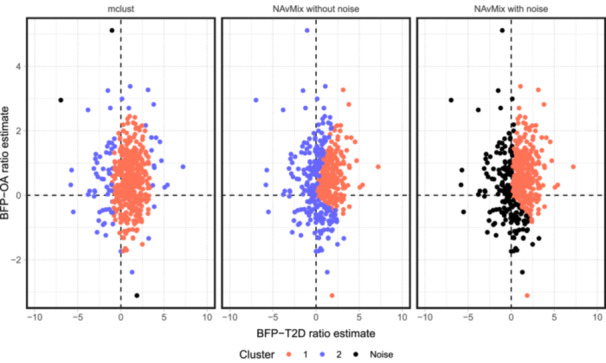
From left to right: The clustering results of the mclust algorithm with an initial noise proportion 5%; the clustering results of the NAvMix method with an initial noise proportion 0; the clustering results of the NAvMix method with an initial noise proportion 5%.

The clustering results generated with mclust and NAvMix are presented in Figure 6. Both methods fail to segregate the variants along the OA‐estimate dimension, as all clusters indicate positive effects, hence might have underestimated the number of clusters, which also appears as an over‐arching feature of the methods in simulations. Even for the T2D‐estimate clustering, their results may be dubious: mclust assigns SNPs in nonadjacent regions with largely opposing estimates into the same cluster (Cluster 2 in blue); NAvMix either labels a large number of SNPs as “junk” if setting a non‐zero initial noise proportion, or does not identify any noise at all with a zero initial proportion. More importantly, for clusters generated by these two methods, variants tend to display substantial within‐cluster heterogeneity in their ratio estimates, which can be a significant concern for causal inference (see Table S15 and S16 in Appendix SF.1).

### 4.3 Biological insights into the variant clusters

To gain insights into the biological mechanisms linking obesity to the T2D‐OA multimorbidity from the variant clusters detected by MR‐AHC, we use an approach similar to the one taken by previous works such as Grant et al. (2022) and Wang et al. (2021). For each of the clusters identified by MR‐AHC, we first map the SNPs in the cluster to genes, then perform gene set enrichment analysis with the mapped genes. Both steps are conducted using the Functional Mapping and Annotation Platform (Watanabe et al., 2017). SNPs are mapped to genes using a three‐way mapping strategy (positional, eQTL and chromatin interactions mapping). The gene set enrichment analysis is to test if the mapped genes are over‐represented in a given predefined gene set which corresponds to a canonical biological pathway or is associated with a phenotype reported from the GWAS catalog (MacArthur et al., 2017). We refer to the latter as the gene‐set Phenome‐wide association analysis (PheWAS), or just “PheWAS” for short. We integrate both lines of evidence from the pathway and PheWAS analyses that can complement or validate each other, to infer the possible biological mechanisms underlying each cluster. See Supporting Information Material S2 for a summary of the enrichment analyses results.

First, it is likely that Cluster 2 (containing 258 SNPs, associated with increasing risk of T2D) is highly pleiotropic. Based on the PheWAS analysis, this cluster is enriched with a large number of phenotypes, double that for Cluster 1 which has the second most (112 vs. 56). These phenotypes fall into a wide range of categories, displaying no clear pattern. The majority of the canonical pathways enriched for this cluster are related to intermediate filament, which might not have a strong direct link with the causal relationship under examination.

Cluster 1 (containing 124 SNPs, indicating increasing risks of both conditions) holds particular significance as it aligns with our primary objective of exploring the multimorbidity of T2D and OA through obesity. The majority of the canonical pathways uniquely enriched for Cluster 1 can be classified into two categories of cellular processes that are closely interconnected: gene expression transcription and cellular responses to stimuli. A significant example in the first category is DNA methylation ($p=8.52\times 10^{-4}$), while in the second category, one of the most significantly enriched pathways is associated with OS ($p=4.05\times 10^{-3}$). For some of the pathways, we can delve deeper into the investigation using readily available GWAS data. As an example, we further inspect the possible common pathway from obesity to the T2D‐OA multimorbidity via OS.

**Figure 7:**
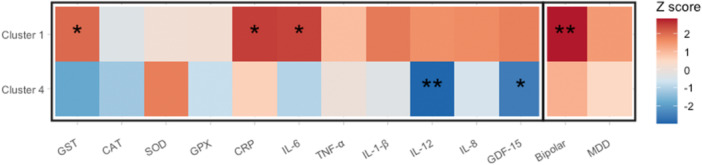
Results of the two‐sample MR estimating the effects of body fat percentage on the 11 oxidative stress biomarkers and 2 psychological disorders using variants in Cluster 1 and Cluster 4 as instruments respectively. Estimates are given by the inverse variance weighted approach, presented in the form of Z‐scores (the ratio of the estimate and the standard error). “*” represents significance at the *p* value 0.05; “**” for the first 11 traits represents significance at 0.05∕11, for the last two traits at 0.05/2.

OS is the imbalance between the production of reactive oxygen species and the counteracting antioxidant defenses in the direction that favors the former, which may lead to tissue injury (Betteridge, 2000). Clinical research has established that obesity can induce systemic OS through various metabolic pathways (Manna & Jain, 2015; Vincent & Taylor, 2006). Moreover, OS is evidenced to exert direct effects on the development of T2D via mechanisms such as decreasing insulin secretion from pancreatic β cells (Furukawa et al., 2017; Matsuoka et al., 1997). It also plays a role in the progression of OA by promoting cartilage degradation (Lepetsos & Papavassiliou, 2016). Herein, we examine the role of OS by performing cluster‐specific MR: we first analyze how BFP predicted by SNPs in Cluster 1 is associated with a variety of OS biomarkers. Then for comparison, we conduct the same analysis on Cluster 4, serving as a counterpart to Cluster 1 due to its relatively benign nature for both conditions, manifesting a protective effect against T2D and a null effect on OA. We select 11 OS biomarkers from diverse categories, which are listed on the *x*‐axis in Figure 7 for the first 11 columns. For details of the selection of the biomarkers and the full form of the abbreviations of the biomarker names, see Appendix SF.2.

We estimate the effect of BFP on each of the biomarkers by two‐sample MR using SNPs in Cluster 1 and Cluster 4 separately. The estimates and standard errors are calculated by the IVW approach. Sensitivity checks by MR‐PRESSO (Verbanck et al., 2018) and MR‐RAPS (Zhao et al., 2020) can be found in Appendix SF.2 Table S17 and S18. Results in Z‐score (the ratio of the estimate and the standard error) are presented in Figure 7. For 8 out of 11 of these OS markers, Cluster 1 is associated with increasing effects, while Cluster 4 is associated with declining effects. For CAT and CRP, Cluster 1 and Cluster 4 have effects in the same direction, but Cluster 1 is either associated with a larger increasing effect (CRP), or a smaller decreasing effect (CAT). The only exception is SOD, on which the increasing effect of Cluster 1 is smaller than that of Cluster 4.

Overall, we can see a clear heterogeneity pattern between Cluster 1 and Cluster 4 in their cluster‐specific effects on the OS biomarkers, which supports that Cluster 1 is associated with an elevated level of OS, while it may be the opposite for Cluster 4. These results align with the existing findings regarding adiposity and OS: higher adiposity is in general associated with elevated OS, but fat patterns featured with a smaller waist‐to‐hip ratio (WHR) may be related to less oxidative damage (Davı et al., 2002; Vincent & Taylor, 2006). This correlation between WHR and OS is observed in Cluster 4, as we will show later that this cluster is associated with a decreasing WHR.

Complementary evidence that might be related to the OS pathway can be found in the PheWAS results for Cluster 1. A notable PheWAS pattern uniquely associated with this cluster is that it is enriched with quite a few psychological disorders. Clinical research has shown that OS is implicated in the development of such disorders, including bipolar disorder (BD) and depression (Salim, 2014), which are both significantly enriched for Cluster 1. We estimate the effects of BFP predicted by variants in respectively Cluster 1 and Cluster 4 on BD and major depressive disorder (MDD) using two‐sample MR. Results are presented in the last two columns in Figure 7. Cluster 1 is associated with increasing risks of both conditions with a significant effect on BD. The effects of Cluster 4, on the other hand, are both insignificant and smaller than those of Cluster 1. These results may suggest a possible direction for exploring the multimorbidity between obesity‐related metabolic conditions and psychological disorders.

It is important to note that there are very likely to be intricate interactions between the pathways involved in the underlying mechanism from obesity to the T2D‐OA multimorbidity. For example, another canonical pathway uniquely enriched for Cluster 1 is related to programmed cell death, or apoptosis. It has been well‐documented that excess OS plays a role in the activation of apoptosis (Redza‐Dutordoir & Averill‐Bates, 2016), and pancreatic β‐cell and chondrocyte loss due to apoptosis are implicated in the development of T2D and OA respectively (Del Carlo & Loeser, 2008; Johnson & Luciani, 2010). Furthermore, quite a few gene expression transcription pathways enriched for Cluster 1 are related to epigenetic processes. Emerging evidence supports the involvement of OS in epigenetic regulation of gene expression such as inducing DNA methylation changes (Franco et al., 2008; Niu et al., 2015). Thus, additional research is warranted to further unravel the exact causal roles of these pathways.

Both Cluster 3 and Cluster 4 exhibit a protective effect against T2D. The most noteworthy PheWAS pattern for these two clusters is that they are both enriched with phenotypes related to fat distribution. This is particularly pronounced for Cluster 4, with 17 out of 42 enriched phenotypes associated with fat patterns including the WHR‐related traits. Also, Cluster 4 has a clear pattern regarding its enriched biological pathways: 13 out of 16 of the pathways are related to ion channel activities. Ion channels are membrane proteins acting as gated pathways for the passage of ions across the cell membranes (Ashcroft, 1999).

To integrate the evidence from the WHR‐enriched PheWAS pattern and the ion‐channel‐enriched pathway pattern into a potential explanation of the protective mechanism against T2D, one possible link may be that Cluster 4 is also enriched with several high‐density lipoprotein cholesterol (HDL‐C) phenotypes. Existing studies have found a negative relationship between WHR and HDL‐C (Ostlund et al., 1990; Wing et al., 1991), i.e. smaller WHR may be associated with higher levels of HDL‐C. Moreover, the connection between HDL‐C levels, ion channel activities, and T2D development might be explained by the primary role of HDL‐C in cholesterol clearance (Schmitz & Grandl, 2009). On one hand, ion channels, such as the β‐cell voltage‐gated calcium channels, are crucial for insulin secretion (Yang & Berggren, 2006). On the other hand, the activity of such channels can be suppressed by excess membrane cholesterol (Levitan et al., 2010). Thus, the depletion of cholesterol facilitated by HDL‐C might positively impact the activity of the ion channels related to insulin secretion. This link is evidenced by previous experimental research on mice, which shows that reduced HDL‐C levels are correlated with impaired glucose‐induced insulin secretion (Xepapadaki et al., 2019). This is because the increased rigidity of the β‐cell membrane due to cholesterol‐enrichment reduces the stimulation of ion channels essential for secreting insulin (Gleason et al., 1991; Xepapadaki et al., 2021).

**Figure 8:**
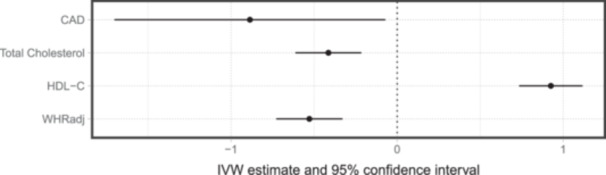
Two‐sample MR results estimating the effects of Cluster 4‐predicted BFP on the waist‐to‐hip ratio adjusted for BMI, HDL‐C, total cholesterol, and coronary artery disease. Estimates are given by the inverse variance weighted approach, presented in the form of the 95% confidence intervals.

To examine the possible protective mechanism against T2D stated above, we conduct two‐sample MR to examine the effects indicated by Cluster 4 on WHR (adjusted for BMI), HDL‐C and total cholesterol levels. The results, shown in Figure 8, are in line with the hypothesized mechanism: this cluster is associated smaller WHRs, higher levels of HDL‐C, lower levels of total cholesterol, and consequently decreasing risk of coronary artery disease (CAD).

## 5 DISCUSSION

In this paper, we adapt the general method of AHC to multi‐sample summary‐data Mendelian randomization. MR‐AHC is a useful tool for interrogating a set of genetic variants to see if they collectively identify a single causal effect, or if it is more plausible that a number of subgroups identify distinct effects driven by different biological mechanisms. The method is of particular interest when the potentially heterogeneous physiological components of the exposure are not known beforehand, or are difficult/expensive to measure. Of special interest is its utility in the multi‐outcome MR setting, where it can be applied to aid in elucidating the unifying causal pathways underlying multimorbidity through a shared risk factor. The common biological mechanisms linking the multimorbid conditions as revealed by the genetic variant clusters may be informative for exploring the therapeutic targets for multimorbidity.

MR‐AHC possesses the features that it does not require prespecifying the number of clusters, and that alongside detecting meaningful clusters it can also identify and label null and junk clusters without an initial specification on the proportion of “noise.” While for hierarchical clustering algorithms, it can be difficult to choose the “optimal” dissimilarity metric, linkage and number of clusters on the dendrogram to yield reliable clustering results, studies in the field of model selection (Andrews, 1999; Apfel & Liang, 2024) provide the theoretical basis for MR‐AHC to ensure highly accurate results. We have adapted the original AHC method to accommodate the varying degrees of uncertainty exhibited in summary‐data estimates. MR‐AHC is also capable of handling outliers in the variant‐specific estimates with our outlier removal procedure. It uses as input the covariance matrix of the ratio estimates, nevertheless, we demonstrate in simulations that it is in general not a significant concern even if the covariances are treated as zero.

In an effort to investigate the disease‐causing mechanisms, a number of approaches has been utilized to categorize genetic variants associated with a specific phenotype, based on their GWAS associations with a range of traits linked to that target phenotype, such as the Bayesian nonnegative matrix factorization clustering method (Udler & Kim, 2018) and NAvMix (Grant et al., 2022). MR‐AHC is motivated similarly, but it is distinctly tailored to the MR framework with primary application rooted in the domain of causal inference. It groups genetic variants based on their causal estimates, which integrates both their associations with the target phenotype (in this context, a common exposure) and their associations with the related traits (herein, downstream outcomes). By the comparison with NAvMix through simulations and the real‐world application, we have shown that MR‐AHC has certain advantages over the association‐based approaches in MR settings, namely an enhanced capacity to identify the patterns of the genetic variants.

It should be noted that all the aforementioned methods for comparison with MR‐AHC assign variants to clusters in a probabilistic (i.e., “soft”) way, while MR‐AHC does the clustering in a deterministic (i.e., “hard”) manner. Although we view this as a strength, some may view its lack of stochasticity as a disadvantage. For this reason, we plan to develop a framework to quantify the sensitivity of MR‐AHC clustering results to small changes in the data and thresholding rule used. Our method currently focuses on the problem of estimating a causal relationship between the shared exposure and the downstream outcomes without accounting for the direct causality between the outcomes. In our application example, various existing evidence supports the absence of direct causality between T2D and OA (Arruda et al., 2023; Khor et al., 2020). However, we show in Appendix SC.2 that even if direct causality exists, our method is still applicable, as the clustering of the variants associated with the common exposure are generally robust to the outcome causality. The challenge then shifts to estimating the direct causal effect of the exposure on a particular outcome while considering other outcome traits as an additional risk factor, or accepting that the original estimates represent total causal effects via the outcome in question. Given this, another potential future extension of our work is to extend the method to the multi‐exposure framework, with the additional flexibility to consider genetic sub‐structure within each exposure.

## AUTHOR CONTRIBUTIONS

Study conception and design: Xiaoran Liang, Nicolas Apfel, Timothy M. Frayling, and Jack Bowden. Methodology development: Xiaoran Liang, Ninon Mounier, Nicolas Apfel, and Jack Bowden. Acquisition of data: Ninon Mounier, Timothy M. Frayling, and Jack Bowden. Designing and conducting the simulations and applied analyses: Xiaoran Liang, Ninon Mounier, and Jack Bowden. Analyzing and interpreting the results: Xiaoran Liang, Ninon Mounier, Sara Khalid, Timothy M. Frayling, and Jack Bowden. Drafting the manuscript: Xiaoran Liang, Ninon Mounier, and Jack Bowden. All authors critically reviewed and edited the manuscript.

## CONFLICT OF INTEREST STATEMENT

Tim Frayling has received funding from GSK and consulted for Sanofi and Boehringer Ingelheim. Jack Bowden is a part time employee of Novo Nordisk, engaged in work unrelated to this project. Other authors declare no conflict of interest.

## Supporting information

Supplementary Information

Supplementary Information

## Data Availability

All data used in the applied analyses in this paper are publicly available. GWAS summary statistics for body fat percentage are obtained from the original study cited in the paper, Supplementary material 1d; the type 2 diabetes data are downloaded from http://diagram-consortium.org/downloads.html; osteoarthritis data are available from https://r8.risteys.finngen.fi/phenocode/M13_ARTHROSIS_INCLAVO; data for GST, CAT, SOD, GPX and CRP are downloaded from https://gwas.mrcieu.ac.uk/; data for IL‐6, IL‐8, IL‐12, IL‐1B, TNF‐A from https://data.bris.ac.uk/data/dataset/c4e3b263f392bb23cd62997d1b14da05; data for GDF‐15 from https://www.ebi.ac.uk/gwas/efotraits/EFO_0009181; data for bipolar disorder and major depression disorder from https://pgc.unc.edu/for-researchers/download-results/; data for waist‐to‐hip ratio from https://portals.broadinstitute.org/collaboration/giant/index.php/GIANT_consortium_data_files#GWAS_2010_WHRadjBMI_Summary_Statistics; data for HDL‐C and total cholesterol from https://csg.sph.umich.edu/willer/public/lipids2013/; data for coronary artery disease from http://www.cardiogramplusc4d.org/data-downloads/. The R code that implements MR‐AHC and that generates the simulation datasets are available on Github: https://github.com/xiaoran-liang/MRAHC.
