# Supplementary material for: Using clustering of genetic variants in Mendelian randomization to interrogate the causal pathways underlying multimorbidity from a common risk factor
Source: Genet Epidemiol. 2024 Aug 13;49(1):e22582. doi: 10.1002/gepi.22582 (PMC11647065; doi:10.1002/gepi.22582)
Supplement: Supplementary file 1 — Supplementary Information [file GEPI-49-0-s002.pdf]

Appendix to "Using clustering of genetic variants in Mendelian randomization to interrogate the causal pathways underlying multimorbidity from a common risk factor"

Xiaoran Liang[a], Ninon Mounier[a], Nicolas Apfel[b], Sara Khalid[c], Timothy M Frayling[a,d], and Jack Bowden[a]

[a]Department of Clinical and Biomedical Sciences, Faculty of Health and Life Sciences, University of Exeter, Exeter, UK

[b]Department of Economics, University of Southampton, Southampton, UK

[c]Centre for Statistics in Medicine, Nuffield Department of Orthopaedics, Rheumatology and Musculoskeletal Sciences, University of Oxford, Oxford, UK

[d]Faculty of Medicine, Department of Genetic Medicine and Development, CMU, Geneva, Switzerland

16 May, 2024

A The weighted squared Euclidean distance

Without loss of generality, consider two variant clusters $\mathcal{S}_k$ and $\mathcal{S}_l$. Suppose the true underlying causal effect from the exposure to the outcome Y_p identified by $\mathcal{S}_k$ is $\beta_{\mathcal{S}_k,p}$. We refer to $\beta_{\mathcal{S}_k,p}$ as the causal estimand of $\mathcal{S}_k$. Similarly, let $\beta_{\mathcal{S}_l,p}$ be the causal estimand of $\mathcal{S}_l$. In all the relevant notations, $p = 1, ..., P$. To test if $\mathcal{S}_k$ and $\mathcal{S}_l$ identify the same causal effects across all outcomes, i.e. to test the null hypothesis

$$H_0 : \boldsymbol{\beta}_{\mathcal{S}_k} = \boldsymbol{\beta}_{\mathcal{S}_l}$$

where $\boldsymbol{\beta}_{\mathcal{S}_k}$, $\boldsymbol{\beta}_{\mathcal{S}_l}$ are P-dimensional vectors of $\beta_{\mathcal{S}_k,p}$ and $\beta_{\mathcal{S}_l,p}$ respectively, we can construct the Wald statistic using the IVW estimates of $\boldsymbol{\beta}_{\mathcal{S}_k}$ and $\boldsymbol{\beta}_{\mathcal{S}_l}$, denoted by $\widehat{\boldsymbol{\beta}}^{IVW}_{\boldsymbol{\mathcal{S}_k}}$ and $\widehat{\boldsymbol{\beta}}^{IVW}_{\boldsymbol{\mathcal{S}_l}}$, as defined in Equation (8), with the p-th entry

$$\widehat{\beta}^{IVW}_{\mathcal{S}_k,p} = \frac{\sum_{j \in S_k} \widehat{\beta}_{jp} w_{jp}}{\sum_{j \in S_k} w_{jp}}.$$

Then the Wald testing statistic for the above null hypothesis is defined as

$$\mathcal{W}_{k,l} = \left(\widehat{\boldsymbol{\beta}}^{IVW}_{\boldsymbol{\mathcal{S}_k}} - \widehat{\boldsymbol{\beta}}^{IVW}_{\boldsymbol{\mathcal{S}_l}}\right)' \widehat{\boldsymbol{Var}}^{-1}_{k,l} \left(\widehat{\boldsymbol{\beta}}^{IVW}_{\boldsymbol{\mathcal{S}_k}} - \widehat{\boldsymbol{\beta}}^{IVW}_{\boldsymbol{\mathcal{S}_l}}\right), \tag{S1}$$

where $\widehat{\boldsymbol{Var}}_{k,l}$ is the covariance matrix of $\left(\widehat{\boldsymbol{\beta}}^{IVW}_{\boldsymbol{\mathcal{S}_k}} - \widehat{\boldsymbol{\beta}}^{IVW}_{\boldsymbol{\mathcal{S}_l}}\right)$. The diagonal entries of $\widehat{\boldsymbol{Var}}_{k,l}$ are the variances of $\left(\widehat{\beta}^{IVW}_{\mathcal{S}_k,p} - \widehat{\beta}^{IVW}_{\mathcal{S}_l,p}\right)$. We assume that all the variants are independent of each other, and all the ratio estimates across different variants are uncorrelated, then we have

$$Var\left(\widehat{\beta}^{IVW}_{\mathcal{S}_k,p} - \widehat{\beta}^{IVW}_{\mathcal{S}_l,p}\right) = Var(\widehat{\beta}^{IVW}_{\mathcal{S}_k,p}) + Var(\widehat{\beta}^{IVW}_{\mathcal{S}_l,p}).$$

The variance of $\widehat{\beta}^{IVW}_{\mathcal{S}_k,p}$ is given by

$$Var(\widehat{\beta}^{IVW}_{\mathcal{S}_k,p}) = \frac{1}{W_{kp}^2} \sum_{j \in \mathcal{S}_k} w_{jp}^2 Var(\widehat{\beta}_{jp}) = \frac{1}{W_{kp}^2} \sum_{j \in \mathcal{S}_k} w_{jp}^2 \frac{1}{w_{jp}} = \frac{1}{W_{kp}}$$

where $W_{kp} = \sum_{j \in \mathcal{S}_k} w_{jp}$ and $w_{jp} = 1/Var(\widehat{\beta}_{jp})$. Then we have

$$Var\left(\widehat{\beta}^{IVW}_{\mathcal{S}_k,p} - \widehat{\beta}^{IVW}_{\mathcal{S}_l,p}\right) = \frac{1}{W_{kp}} + \frac{1}{W_{lp}}. \tag{S2}$$

Thus far, we have the diagonal entries of $\widehat{\boldsymbol{Var}}_{k,l}$ as $\left(\frac{1}{W_{kp}} + \frac{1}{W_{lp}}\right)$ with $p = 1, ..., P$. Now we need to derive the covariance term between $\left(\widehat{\beta}^{IVW}_{\mathcal{S}_k,i} - \widehat{\beta}^{IVW}_{\mathcal{S}_l,i}\right)$ and $\left(\widehat{\beta}^{IVW}_{\mathcal{S}_k,r} - \widehat{\beta}^{IVW}_{\mathcal{S}_l,r}\right)$, with $i, r \in \{1, ..., P\}$, $i \neq r$. Start with

$$\begin{aligned}
&cov\left(\left(\widehat{\beta}^{IVW}_{\mathcal{S}_k,i} - \widehat{\beta}^{IVW}_{\mathcal{S}_l,i}\right), \left(\widehat{\beta}^{IVW}_{\mathcal{S}_k,r} - \widehat{\beta}^{IVW}_{\mathcal{S}_l,r}\right)\right) \\
&= cov\left(\widehat{\beta}^{IVW}_{\mathcal{S}_k,i}, \widehat{\beta}^{IVW}_{\mathcal{S}_k,r}\right) + cov\left(\widehat{\beta}^{IVW}_{\mathcal{S}_l,r}, \widehat{\beta}^{IVW}_{\mathcal{S}_l,r}\right) \\
&= cov\left(\frac{\sum_{j\in\mathcal{S}_k} \widehat{\beta}_{ji} w_{ji}}{\sum_{j\in\mathcal{S}_k} w_{ji}}, \frac{\sum_{j\in\mathcal{S}_k} \widehat{\beta}_{jr} w_{jr}}{\sum_{j\in\mathcal{S}_k} w_{jr}}\right) + cov\left(\frac{\sum_{j\in\mathcal{S}_l} \widehat{\beta}_{ji} w_{ji}}{\sum_{j\in\mathcal{S}_l} w_{ji}}, \frac{\sum_{j\in\mathcal{S}_l} \widehat{\beta}_{jr} w_{jr}}{\sum_{j\in\mathcal{S}_l} w_{jr}}\right)
\end{aligned} \tag{S3}$$

For the first term, we have

$$\begin{aligned}
&cov\left(\frac{\sum_{j\in\mathcal{S}_k} \widehat{\beta}_{ji} w_{ji}}{\sum_{j\in\mathcal{S}_k} w_{ji}}, \frac{\sum_{j\in\mathcal{S}_k} \widehat{\beta}_{jr} w_{jr}}{\sum_{j\in\mathcal{S}_k} w_{jr}}\right) \\
&\approx \frac{1}{W_{ki} W_{kr}} cov\left(\sum_{j\in\mathcal{S}_k} \widehat{\beta}_{ji} w_{ji}, \sum_{j\in\mathcal{S}_k} \widehat{\beta}_{jr} w_{jr}\right) \\
&\approx \frac{1}{W_{ki} W_{kr}} \sum_{j\in\mathcal{S}_k} w_{ji} w_{jr} cov(\widehat{\beta}_{ji}, \widehat{\beta}_{jr})
\end{aligned} \tag{S4}$$

Now we need to derive $cov(\widehat{\beta}_{ji}, \widehat{\beta}_{jr})$. By definition, we have

$$cov(\widehat{\beta}_{ji}, \widehat{\beta}_{jr}) = cov\left(\frac{\widehat{\Gamma}_{ji}}{\widehat{\gamma}_j}, \frac{\widehat{\Gamma}_{jr}}{\widehat{\gamma}_j}\right) \approx \frac{cov\left(\widehat{\Gamma}_{ji}, \widehat{\Gamma}_{jr}\right)}{\widehat{\gamma}_j^2}$$

Consider a case where we obtain $\left(\widehat{\Gamma}_{ji}, \widehat{\Gamma}_{jr}, \widehat{\gamma}_j\right)$ from three samples with *i.i.d.* individuals, we have

$$Y_i = G_j \Gamma_{ji} + \epsilon_{ji}$$

$$Y_r = G_j\Gamma_{jr} + \epsilon_{jr}$$

where G_j is independent with ϵ_{ji} and ϵ_{jr}. Assume Y_i, Y_r and G_j all have mean 0, and G_j has variance equal to 1. Let N_i, N_r and N_o denote the sample size for Y_i, Y_r and their overlap respectively. Following the derivation in Wang et al. (2021) Appendix S2, we have:

$$\widehat{\Gamma}_{ji} = \frac{\widehat{cov_{N_i}(Y_i, G_j)}}{\widehat{cov_{N_i}(G_j, G_j)}} = \widehat{cov_{N_i}(Y_i, G_j)} = \frac{1}{N_i}\sum_{m=1}^{N_i} Y_{im}G_{jm}$$

$$\widehat{\Gamma}_{jr} = \frac{\widehat{cov_{N_r}(Y_r, G_j)}}{\widehat{cov_{N_r}(G_j, G_j)}} = \widehat{cov_{N_r}(Y_r, G_j)} = \frac{1}{N_r}\sum_{m=1}^{N_r} Y_{rm}G_{jm}.$$

Then

$$\begin{aligned}
cov\left(\widehat{\Gamma}_{ji}, \widehat{\Gamma}_{jr}\right) &= cov\left(\frac{1}{N_i}\sum_{m=1}^{N_i} Y_{im}G_{jm}, \frac{1}{N_r}\sum_{m=1}^{N_r} Y_{rm}G_{jm}\right) \\
&= \frac{1}{N_iN_r} cov\left(\sum_{m=1}^{N_i} Y_{im}G_{jm}, \sum_{m=1}^{N_r} Y_{rm}G_{jm}\right) \\
&= \frac{1}{N_iN_r} cov\left(\sum_{m=1}^{N_o} Y_{im}G_{jm}, \sum_{m=1}^{N_o} Y_{rm}G_{jm}\right) \\
&= \frac{N_o}{N_iN_r} cov\left(Y_iG_j, Y_rG_j\right) = \frac{N_o}{N_iN_r} cov\left(G_r^2\Gamma_{ji} + G_j\epsilon_{ji}, G_j^2\Gamma_{jr} + G_j\epsilon_{jr}\right) \\
&= \frac{N_o}{N_iN_r}\left(\Gamma_{ji}\Gamma_{jr}Var(G_j^2) + \Gamma_{ji}cov(G_j^2, G_j\epsilon_{ji}) + \Gamma_{jr}cov(G_j^2, G_j\epsilon_{jr}) + cov(G_j\epsilon_{ji}, G_j\epsilon_{jr})\right)
\end{aligned}$$

Following the argument in Wang et al. (2021), for most the variants, their individual genetic effects Γ_{ji} and Γ_{jr} are very small, so approximately

$$\begin{aligned}
cov\left(\widehat{\Gamma}_{ji}, \widehat{\Gamma}_{jr}\right) &\approx \frac{N_o}{N_iN_r} cov\left(G_j\epsilon_{ji}, G_j\epsilon_{jr}\right) \\
&= \frac{N_o}{N_iN_r}\left(E(G_j^2\epsilon_{ji}\epsilon_{ji}) - E(G_j\epsilon_{jr})E(G_j\epsilon_{jr})\right)
\end{aligned}$$

As

$$E(G_j\epsilon_{ji}) = E_G\left(E(G_j\epsilon_{ji}|G_j)\right) = E_G\left(G_jE(\epsilon_{ji}|G_j)\right) = 0,$$

then

$$cov\left(\widehat{\Gamma}_{ji}, \widehat{\Gamma}_{jr}\right) = \frac{N_o}{N_iN_r}E(G_j^2\epsilon_{ji}\epsilon_{jr})$$

Because G_j and $\epsilon_{ji}, \epsilon_{jr}$ are independent, then

$$\begin{aligned} cov\left(\widehat{\Gamma}_{ji}, \widehat{\Gamma}_{jr}\right) &= \frac{N_o}{N_iN_r}E(G_j^2\epsilon_{ji}\epsilon_{jr}) = \frac{N_o}{N_iN_r}E(G_j^2)E(\epsilon_{ji}\epsilon_{jr}) \\ &= \frac{N_o}{N_iN_r}Var(G_j^2)cov(\epsilon_{ji}\epsilon_{jr}) = \frac{N_o}{N_iN_r}cov(\epsilon_{ji}\epsilon_{jr}) \\ &= \frac{N_o}{N_iN_r}cov(Y_i - G_j\Gamma_{ji}, Y_r - G_j\Gamma_{jr}) \\ &\approx \frac{N_o}{N_iN_r}cov(Y_i, Y_r) \end{aligned}$$

By definition, the correlation between $\widehat{\Gamma}_{ji}$ and $\widehat{\Gamma}_{jr}$, denoted by ρ_{jir}, is

$$\rho_{jir} = \frac{cov\left(\widehat{\Gamma}_{ji}, \widehat{\Gamma}_{jr}\right)}{se\left(\widehat{\Gamma}_{ji}\right)se\left(\widehat{\Gamma}_{jr}\right)}.$$

By

$$\begin{aligned} Var(\widehat{\Gamma}_{ji}) &= Var(\frac{1}{N_i}\sum_{m=1}^{N_i}Y_{im}G_{jm}) = \frac{1}{N_i}Var(Y_iG_j) \\ &= \frac{1}{N_i}Var(G_j^2\widehat{\Gamma}_{ji} + G_j\epsilon_{ji}) \\ &\approx \frac{1}{N_i}Var(G_j\epsilon_{ji}) = \frac{1}{N_i}Var(G_j)Var(\epsilon_{ji}) \\ &= \frac{1}{N_i}Var(\epsilon_{ji}) \approx \frac{1}{N_i}Var(Y_i), \end{aligned}$$

we have

$$\rho_{jir} = \frac{cov\left(\widehat{\Gamma}_{ji}, \widehat{\Gamma}_{jr}\right)}{se\left(\widehat{\Gamma}_{ji}\right) se\left(\widehat{\Gamma}_{jr}\right)} = \frac{N_o}{\sqrt{N_i N_r}} \frac{cov(Y_i, Y_r)}{se\left(Y_i\right) se\left(Y_r\right)} = \frac{N_o}{\sqrt{N_i N_r}} corr(Y_i, Y_r),$$

where $corr(Y_i Y_r)$ is the phenotypic correlation between the two outcome traits Y_i and Y_r. Since it is the same across $j = 1, ..., J$, we omit the subscript j. Given ρ_{ir}, we have

$$cov\left(\widehat{\Gamma}_{ji}, \widehat{\Gamma}_{jr}\right) = \rho_{ir} se\left(\widehat{\Gamma}_{ji}\right) se\left(\widehat{\Gamma}_{jr}\right).$$

Then

$$cov(\widehat{\beta}_{ji}, \widehat{\beta}_{jr}) = cov\left(\frac{\widehat{\Gamma}_{ji}}{\widehat{\gamma}_j}, \frac{\widehat{\Gamma}_{jr}}{\widehat{\gamma}_j}\right) \approx \frac{cov\left(\widehat{\Gamma}_{ji}, \widehat{\Gamma}_{jr}\right)}{\widehat{\gamma}_j^2} = \frac{\rho_{ir} se\left(\widehat{\Gamma}_{ji}\right) se\left(\widehat{\Gamma}_{jr}\right)}{\widehat{\gamma}_j^2}.$$

Plug into Equation (S4), we have

$$\begin{aligned}
&cov\left(\frac{\sum_{j\in\mathcal{S}_k} \widehat{\beta}_{ji} w_{ji}}{\sum_{j\in\mathcal{S}_k} w_{ji}}, \frac{\sum_{j\in\mathcal{S}_k} \widehat{\beta}_{jr} w_{jr}}{\sum_{j\in\mathcal{S}_k} w_{jr}}\right) \\
&= \frac{1}{W_{ki} W_{kr}} \sum_{j\in\mathcal{S}_k} w_{ji} w_{jr} cov(\widehat{\beta}_{ji}, \widehat{\beta}_{jr}) \\
&= \frac{1}{W_{ki} W_{kr}} \sum_{j\in\mathcal{S}_k} w_{ji} w_{jr} \rho_{ir} se\left(\widehat{\Gamma}_{ji}\right) se\left(\widehat{\Gamma}_{jr}\right) / \widehat{\gamma}_j^2 \\
&= \frac{\rho_{ir}}{W_{ki} W_{kr}} \sum_{j\in\mathcal{S}_k} \frac{\widehat{\gamma}_j^2}{se\left(\widehat{\Gamma}_{ji}\right) se\left(\widehat{\Gamma}_{jr}\right)}
\end{aligned}$$

as $w_{ji} = \widehat{\gamma}_j^2 / se\left(\widehat{\Gamma}_{ji}\right)^2$. Plug this into Equation (S3), we have

$$\begin{aligned}
&cov\left(\left(\widehat{\beta}^{IVW}_{\mathcal{S}_k,i} - \widehat{\beta}^{IVW}_{\mathcal{S}_l,i}\right), \left(\widehat{\beta}^{IVW}_{\mathcal{S}_k,r} - \widehat{\beta}^{IVW}_{\mathcal{S}_l,r}\right)\right) \\
&= cov\left(\frac{\sum_{j\in\mathcal{S}_k} \widehat{\beta}_{ji} w_{ji}}{\sum_{j\in\mathcal{S}_k} w_{ji}}, \frac{\sum_{j\in\mathcal{S}_k} \widehat{\beta}_{jr} w_{jr}}{\sum_{j\in\mathcal{S}_k} w_{jr}}\right) + cov\left(\frac{\sum_{j\in\mathcal{S}_l} \widehat{\beta}_{ji} w_{ji}}{\sum_{j\in\mathcal{S}_l} w_{ji}}, \frac{\sum_{j\in\mathcal{S}_l} \widehat{\beta}_{jr} w_{jr}}{\sum_{j\in\mathcal{S}_l} w_{jr}}\right) \\
&= \frac{\rho_{ir}}{W_{ki} W_{kr}} \sum_{j\in\mathcal{S}_k} \frac{\widehat{\gamma}_j^2}{se\left(\widehat{\Gamma}_{ji}\right) se\left(\widehat{\Gamma}_{jr}\right)} + \frac{\rho_{ir}}{W_{li} W_{lr}} \sum_{j\in\mathcal{S}_l} \frac{\widehat{\gamma}_j^2}{se\left(\widehat{\Gamma}_{ji}\right) se\left(\widehat{\Gamma}_{jr}\right)}.
\end{aligned} \tag{S5}$$

To this end, we obtain $\widehat{\boldsymbol{Var}}_{k,l}$ with the entry on the i-th row and r-th column defined in (S5). When $i = r$, S5 reduces to the variance term defined in S2.

We can see that the covariance matrix $\widehat{\boldsymbol{Var}}_{k,l}$ in the Wald statistic (S1) is equivalent to the weighted squared Euclidean distance $\mathcal{D}_{k,l}$ defined in (9). Therefore, merging the closest two clusters measured by the distance is equivalent to merging two clusters that have the highest similarity in their cluster-specific causal effects.

The covariance terms of $\mathcal{D}_{k,l}$ depend on both the extent of overlap between the Y_i and Y_r samples, and their phenotypic correlation. If the variant-outcome associations are measured with independent samples, and/or the outcome traits are uncorrelated, then all the covariance terms are zero. If the correlation is non-zero, according to Bulik-Sullivan et al. (2015), ρ_{ir} can be estimated from the intercept of the LD score regression:

$$E[z_{ji}z_{jr}] = \frac{\sqrt{N_i N_r}\rho_g}{M} l_j + \frac{\rho_Y N_o}{\sqrt{N_i N_r}}$$

where z_{ji}, z_{jr} are the z-scores, ρ_g is the genetic covariance, M is the number of variants, and l_j is the LD score, ρ_Y is the phenotypic correlation between Y_i and Y_r.

B The threshold p-value of Cochran's Q test

We consider the case of one outcome and omit the subscript p representing the p-th outcome in the following notations. In the downward testing procedure, define Q_{fg} to be the Q statistic associated with cluster $\mathcal{S}_g$ at step f. Also define T_{fg} to be the $(1-\zeta)$ threshold of a χ^2 distribution on $|\mathcal{S}_g| - 1$ degrees of freedom where $|\mathcal{S}_g|$ is the number of variants in cluster $\mathcal{S}_g$. Following a similar strategy as in Windmeijer et al. (2021) and Apfel and Liang (2021), we show that with $\zeta = 0.1/\log(n)$ where n is the size of the sample from which we obtain the variant-outcome summary statistics $\left\{\widehat{\Gamma}_j, se\left(\widehat{\Gamma}_j\right)\right\}_{j=1}^{J}$, both the Type I and Type II error of the test associated with Q_{fg} goes to 0 as $n \to \infty$.

First, we establish the asymptotic property of Q_{fg} as $n \to \infty$:

1. $Q_{fg} \xrightarrow{d} \chi^2_{|\mathcal{S}_g|-1}$ if all variants in $\mathcal{S}_g$ identify the same causal effect (Greco M et al., 2015).

2. $Q_{fg} = O_p(n)$ if variants in $\mathcal{S}_g$ identify different causal effects.

The proof for Point 2 is as follows (all the summations are for $j \in \mathcal{S}_g$):

Based on the definition of $\widehat{\beta}_j$ and w_j, by the continuous mapping theorem, we haves

$$plim(\widehat{\beta}_j) = plim(\widehat{\Gamma}_j/\widehat{\gamma}_j) = \Gamma_j/\gamma_j = \beta_j,$$

$$plim(w_j) = plim\left(1/\widehat{Var(\widehat{\beta}_j)}\right) = plim\left(\frac{\widehat{\gamma}_j^2}{se\left(\widehat{\Gamma}_j\right)^2}\right) = \frac{n\gamma_j^2}{\sigma_{Yj}^2}.$$

Then

$$\begin{aligned}
plim(Q_{fg}) &= plim\left(\sum w_j\left(\widehat{\beta}_j - \frac{\sum w_j\widehat{\beta}_j}{\sum w_j}\right)^2\right) = \sum plim\left(w_j\left(\widehat{\beta}_j - \frac{\sum w_j\widehat{\beta}_j}{\sum w_j}\right)^2\right) \\
&= \sum\left(\frac{n\gamma_j^2}{\sigma_{Yj}^2} \times plim\left(\widehat{\beta}_j - \frac{\sum w_j\widehat{\beta}_j}{\sum w_j}\right)^2\right) = \sum\left(\frac{n\gamma_j^2}{\sigma_{Yj}^2} \times plim\left(\frac{\widehat{\beta}_j\sum w_j - \sum w_j\widehat{\beta}_j}{\sum w_j}\right)^2\right) \\
&= \sum\left(\frac{n\gamma_j^2}{\sigma_{Yj}^2} \times plim\left(\frac{\widehat{\beta}_j\sum\frac{n\gamma_j^2}{\sigma_{Yj}^2} - \sum\frac{n\gamma_j^2}{\sigma_{Yj}^2}\widehat{\beta}_j}{\sum\frac{n\gamma_j^2}{\sigma_{Yj}^2}}\right)^2\right) \\
&= \sum\left(\frac{n\gamma_j^2}{\sigma_{Yj}^2} \times plim\left(\frac{\widehat{\beta}_j\sum\frac{\gamma_j^2}{\sigma_{Yj}^2} - \sum\frac{\gamma_j^2}{\sigma_{Yj}^2}\widehat{\beta}_j}{\sum\frac{\gamma_j^2}{\sigma_{Yj}^2}}\right)^2\right) \\
&= \sum\left(\frac{n\gamma_j^2}{\sigma_{Yj}^2} \times \left(\frac{\beta_j\sum\frac{\gamma_j^2}{\sigma_{Yj}^2} - \sum\frac{\gamma_j^2}{\sigma_{Yj}^2}\beta_j}{\sum\frac{\gamma_j^2}{\sigma_{Yj}^2}}\right)^2\right).
\end{aligned}$$

For $\beta_j\sum\frac{\gamma_j^2}{\sigma_{Yj}^2} - \sum\frac{\gamma_j^2}{\sigma_{Yj}^2}\beta_j = \sum_{i\neq j}(\beta_j - \beta_i)\frac{\gamma_i^2}{\sigma_{Yi}^2}$, as variants in $\mathcal{S}_g$ identify different effects, there is at least one $\beta_j - \beta_i \neq 0$. Therefore, we have $\beta_j\sum\frac{\gamma_j^2}{\sigma_{Yj}^2} - \sum\frac{\gamma_j^2}{\sigma_{Yj}^2}\beta_j \neq 0$ considering the general case where the non-zero terms do not cancel out. It follows that

$$plim(Q_{fg}) = \sum \left(\frac{n\gamma_j^2}{\sigma_{Yj}^2} \times \left(\frac{\beta_j \sum \frac{\gamma_j^2}{\sigma_{Yj}^2} - \sum \frac{\gamma_j^2}{\sigma_{Yj}^2}\beta_j}{\sum \frac{\gamma_j^2}{\sigma_{Yj}^2}} \right)^2 \right) = \sum O_p(n) = O_p(n).$$

If T_{fg} satisfies $T_{fg} \to \infty$, $T_{fg} = o(n)$, it follows that

$$\lim_{n\to\infty} P\left(T_{fg} < Q_{fg}\right) = 1,$$

hence the Q test is rejected if variants in $\mathcal{S}_g$ identify different effects, and

$$\lim_{n\to\infty} P\left(Q_{fg} < T_{fg}\right) = 1,$$

if variants in $\mathcal{S}_g$ identify the same effect. In this way, both the Type I and Type II error of Cochran's Q test goes to 0 as $n \to \infty$. Andrews (1999) show that T_{fg} that satisfies $T_{fg} \to \infty$ and $T_{fg} = o(n)$ can be translated into the threshold p-value that satisfies $\zeta \to 0$ and $log(\zeta) = o(n)$. We then set $\zeta = 0.1/log(n)$ following the suggestion of Belloni et al. (2012). When there are multiple outcomes, the Q statistic defined in (10) is a direct extension of the following single-outcome Q:

$$Q_{fg} = \sum_{j\in\mathcal{S}_{fg}} w_j(\hat{\beta}_j - \hat{\beta}^{IVW}_{\mathcal{S}_{fg}})^2 = \left(\widehat{\boldsymbol{\beta}}_{\mathcal{S}_{fg}} - \boldsymbol{\iota}_k\hat{\beta}^{IVW}_{\mathcal{S}_{fg}}\right)' Var(\widehat{\boldsymbol{\beta}}_{fg})^{-1} \left(\widehat{\boldsymbol{\beta}}_{\mathcal{S}_{fg}} - \boldsymbol{\iota}_k\hat{\beta}^{IVW}_{\mathcal{S}_{fg}}\right),$$

where $\widehat{\boldsymbol{\beta}}_{\mathcal{S}_{fg}}$ is replaced by the $P \times |\mathcal{S}_{fg}|$ length vector $\boldsymbol{\mathcal{B}}_{\mathcal{S}_{fg}}$ combing ratio estimates across all P outcomes, and $\hat{\beta}^{IVW}_{\mathcal{S}_{fg}}$ is adjusted as the IVW estimates for each outcome. In this case, the degrees of freedom would be $P \times (|\mathcal{S}_{fg}| - 1)$.

C Further details of the model setup

C.1 Horizontal pleiotropy

In this section we discuss how different settings of horizontal pleiotropy can affect the clustering phenomenon amongst the genetic variants. Following the simple case where each individual variant G_j only instruments one exposure sub-component X_k, the variant-specific ratio estimand is given by

$$\beta_{jp} = \frac{\theta_{kp}\gamma_{kj} + \alpha_{jp}}{\gamma_{kj}} = \theta_{kp} + \frac{\alpha_{jp}}{\gamma_{kj}} = \theta_{kp} + \frac{\psi_{jp} + q_{yp}\eta_j}{\delta_{kj} + q_{xk}\eta_j}. \tag{S6}$$

In this equation, ψ_{jp} represents the direct pleiotropic effect from G_j on the outcome Y_p that is independent from the effects from G_j on X_k. G_j can also exert indirect effects on Y_p and X_k via the common confounder U, denoted by $q_{yp}\eta_j$ and $q_{xk}\eta_j$ respectively, which represents the correlated pleiotropy.

Now we show how different settings of pleiotropy can result in different variant-specific ratio estimands, and subsequently affect the clustering identification. We use a illustrative example, depicted in Figure S1: suppose we have two sub-components X_1 and X_2 in the exposure that exert heterogeneous causal effects on the outcomes. For ease of illustration we use a single notation Y for the outcomes. U is the uncontrolled confounder between the exposure and outcomes. Serving as instruments for each sub-exposure, we have two variant clusters. Cluster 1 is set to be the pleiotropic cluster in the sense that variants in this cluster affects Y via pathways not through X_1 (directly and/or through U). Cluster 2 is the non-pleiotropic cluster and variants in this cluster only exert effects on Y via X_2.

Since we impose the IV relevance assumption, we do not consider the cases where both $\delta_{kj} = 0$ and $\eta_j = 0$. For variants in Cluster 2, we have $\psi_{jp} = \eta_j = 0$ and all their ratio estimands equal to $\boldsymbol{\theta_2}$. Based on the definition that "variants identify the same causal effect (i.e. have the same ratio estimand) belong to the same cluster", variants in Cluster

2 that affect X and Y through the same biological pathway also belong to the same cluster statistically.

For variants in Cluster 1, there can be some complications depending on the parameter specification. Let G_j denote an individual variant in Cluster 1. When there is only correlated pleiotropy and no direct effects from G_j to Y and X_1 (the top-left panel in Figure S1), we have $\psi_j = 0$, $\delta_{1j} = 0$ and $\eta_j \neq 0$. In this case, following Equation (S6), the ratio estimand of G_j on outcome Y_p is given by (we fix $q_{yp} \neq 0$ and $q_{xk} \neq 0$):

$$\beta_{jp} = \theta_{1p} + \frac{q_{yp}\eta_j}{q_{x1}\eta_j} = \theta_{1p} + \frac{q_{yp}}{q_{x1}}.$$

It can be seen that this ratio estimand does not depend on the variant index j, i.e. all the variants in Cluster 1 have the same ratio estimand. Therefore, all the pleiotropic variants belong to the same cluster.

Next consider the scenario where there is still correlated pleiotropy only, but the pleiotropic variants also exert direct effects on X_1 (the top-right panel in Figure S1). In this case, we have $\psi_j = 0$, $\delta_{1j} \neq 0$ and $\eta_j \neq 0$. The ratio estimand for G_j in Cluster 1 is

$$\beta_{jp} = \theta_{1p} + \frac{q_{yp}\eta_j}{\delta_{1j} + q_{x1}\eta_j},$$

which now becomes variant-specific. This means that while variants in Cluster 1 affect the exposure and outcome through the same biological pathway, the quantities they identify as causal effects can be different if they have different direct effects on the confounder and exposure (i.e. different values of η_j and δ_{1j}). Therefore, in this scenario, the pleiotropic variants may not belong to the same cluster despite of affecting the phenotypes via the same pathway.

Following the same argument, we can derive the ratio estimand for G_j in Cluster 1 under the model setup with only uncorrelated pleiotropy ($\psi_j \neq 0$, $\delta_{1j} \neq 0$ and $\eta_j = 0$,

depicted in the bottom-left panel in Figure S1) as

$$\beta_{jp} = \theta_{1p} + \frac{\psi_{jp}}{\delta_{1j}}.$$

For the model setup with both correlated and uncorrelated pleiotropy (bottom-right panel in Figure S1), with pleiotropic variants exerting direct effects on the exposure, the ratio estimand for G_j in Cluster 1 is

$$\beta_{jp} = \theta_{1p} + \frac{\psi_{jp} + q_{yp}\eta_j}{\delta_{1j} + q_{x1}\eta_j};$$

and without direct effects of G_j on X_1, the ratio estimand is

$$\beta_{jp} = \theta_{1p} + \frac{\psi_{jp} + q_{yp}\eta_j}{q_{x1}\eta_j} = \theta_{1p} + \frac{q_{yp}}{q_{x1}} + \frac{\psi_{jp}}{q_{x1}\eta_j}.$$

In all these scenarios, the pleiotropic variants may have different ratio estimands, hence may not belong to the same cluster (unless some of them by chance have the same value of η_j, δ_{1j} and ψ_{jp}).

In the main simulation studies presented in Section 3 and Appendix D, we examine the performance of the methods under the model setup with only correlated pleiotropy and no direct effects from the pleiotropic variants to the exposure, thus pleiotropic variants have the same ratio estimand. We present further simulations in Appendix E.2 concerning the setup where pleiotropic variants do not have the same ratio estimand.

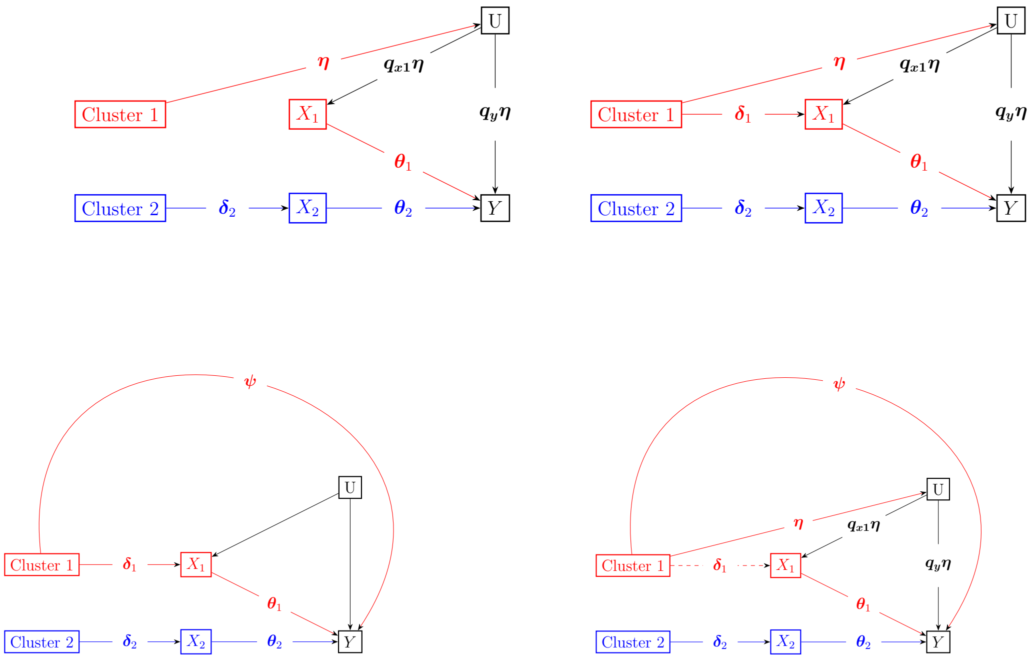

Figure S1: (Top-left) Directed acyclic graphs (DAG) of the model setup with only correlated pleiotropy. The absence of arrows from Cluster 1 to X_1 and Y represents zero direct effects; (Top-right) Directed acyclic graphs (DAG) of the model setup with only correlated pleiotropy, with variants in Cluster 1 exerting direct effects on X_1; (Bottom-left) Directed acyclic graphs (DAG) of the model setup with only uncorrelated pleiotropy; (Bottom-right) Directed acyclic graphs (DAG) of the model setup with both correlated and uncorrelated pleiotropy. The dashed line between Cluster 1 and X_1 denotes the possible absence of direct effects of Cluster 1 on X_1.

C.2 Direct causality between outcomes

We illustrate how the direct causality between outcomes impacts the clustering of the variants associated with the exposure. Consider the model with two outcomes and three exposure sub-components, as depicted in the DAG in Figure S2, in which we add direct causal effects between Y_1 and Y_2: λ_1 denotes the direct effect of Y_1 on Y_2, and λ_2 denotes the direct effect of Y_2 on Y_1. G_1 represents the pleiotropic variant. Maintain the assumption that all the variants are independent with each other. For ease of illustration, we assume that there is one variant in each cluster.

We then have

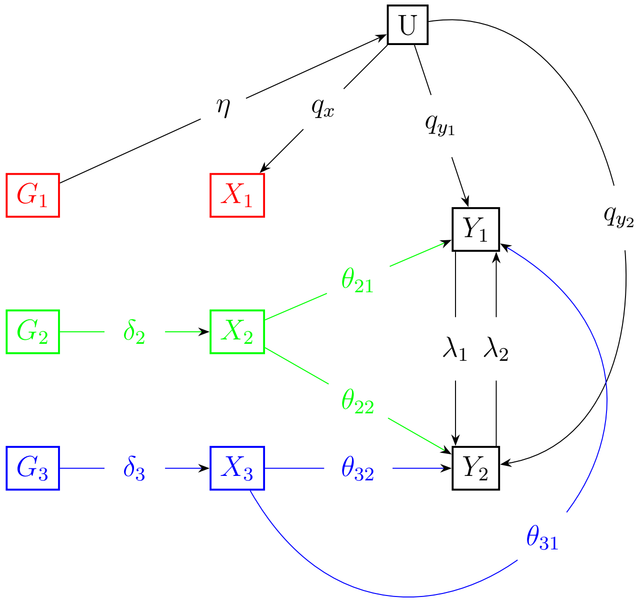

Figure S2: DAG of the extended model incorporating direct causality between outcomes.

$$
\begin{aligned}
U &= \eta G_1 + \epsilon_U, \\
X &= X_1 + X_2 + X_3 \\
&= q_x \eta G_1 + \delta_2 G_2 + \delta_3 G_3 + \epsilon_x, \\
Y_1 &= q_{y_1} U + \theta_{21} X_2 + \theta_{31} X_3 + \lambda_2 Y_2 + \epsilon_{y_1}, \\
Y_2 &= q_{y_2} U + \theta_{22} X_2 + \theta_{32} X_3 + \lambda_1 Y_1 + \epsilon_{y_2}.
\end{aligned}
$$

where G is uncorrelated with all the error terms. To express Y_1 and Y_2 only in G, we need to solve the following equation system:

$$
\begin{aligned}
Y_1 &= q_{y_1} U + \theta_{21} X_2 + \theta_{31} X_3 + \lambda_2 Y_2 + \epsilon_{y_1}, \\
Y_2 &= q_{y_2} U + \theta_{22} X_2 + \theta_{32} X_3 + \lambda_1 Y_1 + \epsilon_{y_2}.
\end{aligned}
$$

Plug U and X expressed in G into the above equations, and solve the system, we then obtain

$$Y_1 = \frac{q_{y_1}\eta + \lambda_2 q_{y_2}\eta}{1-\lambda_1\lambda_2}G_1 + \frac{(\lambda_2\theta_{22}+\theta_{21})\delta_2}{1-\lambda_1\lambda_2}G_2 + \frac{(\lambda_2\theta_{32}+\theta_{31})\delta_3}{1-\lambda_1\lambda_2}G_3 + \xi_1,$$
$$Y_2 = \frac{q_{y_2}\eta + \lambda_1 q_{y_1}\eta}{1-\lambda_1\lambda_2}G_1 + \frac{(\lambda_1\theta_{21}+\theta_{22})\delta_2}{1-\lambda_1\lambda_2}G_2 + \frac{(\lambda_1\theta_{31}+\theta_{32})\delta_3}{1-\lambda_1\lambda_2}G_3 + \xi_2,$$

where G is uncorrelated with the implicitly defined error terms ξ_1, ξ_2. As X can be expressed in G as follows:

$$X = q_x\eta G_1 + \delta_2 G_2 + \delta_3 G_3 + \epsilon_x,$$

then the causal effects identified by each variant are:

$$\begin{aligned} G_1 &: \left(\frac{q_{y_1}+\lambda_2 q_{y_2}}{(1-\lambda_1\lambda_2)q_x}, \frac{q_{y_2}+\lambda_1 q_{y_1}}{(1-\lambda_1\lambda_2)q_x}\right) \\ G_2 &: \left(\frac{\lambda_2\theta_{22}+\theta_{21}}{1-\lambda_1\lambda_2}, \frac{\lambda_1\theta_{21}+\theta_{22}}{1-\lambda_1\lambda_2}\right) \\ G_3 &: \left(\frac{\lambda_2\theta_{32}+\theta_{31}}{1-\lambda_1\lambda_2}, \frac{\lambda_1\theta_{31}+\theta_{32}}{1-\lambda_1\lambda_2}\right). \end{aligned} \tag{S7}$$

Since G is uncorrelated with all the error terms in the aforementioned equations, and all variants are independent with each other, the variant-exposure and variant-outcome associations can be estimated consistently by regressing X and Y on each G respectively. It follows that the ratio estimates of the variants converges to the quantities defined in (S7) as the sample size tends to infinity. Therefore, taking the outcome causality into account, variants impacting the exposure and outcomes through different pathways can still be grouped into the corresponding clusters, as only the variants belonging to the same pathway identify the same effects. However, in this case, the effects identified by each cluster would be the total effects incorporating the outcome causality, rather than the direct causal effects from the exposure to outcomes. Further work is required to address this issue.

D Further simulation details

D.1 Simulation designs

For all the simulation designs, we simulate two/three-sample summary data based on the data generating process defined in Model (1)-(4) with all sample sizes equal to $N = 60,000$. Here we fix $\psi_j = 0$. We assume that G_j, X and Y_p are normalized with $Var(G_j) = Var(X) = Var(Y_p) = 1$ and $\mathbb{E}[G_j] = \mathbb{E}[X] = \mathbb{E}[Y_p] = 0$. We also assume that the covariances between the variants are 0, $Cov(G_j, G_i) = 0$ for $i \neq j$. According to the model, the variant-exposure and variant-outcome summary statistics are generated in the following way:

$$\widehat{\beta}_{Xj} = \sum_{k=1}^{K} \delta_{kj} + \eta_j \sum_{k=1}^{K} q_{xk} + N(0, 1/N),$$
$$\widehat{\beta}_{Yjp} = \sum_{k=1}^{K} \theta_{kp}\delta_{kj} + \eta_j (\sum_{k=1}^{K} \theta_{kp} q_{xk} + q_{yp}) + e_{Ypj},$$

for $j = 1, ..., J$ with $J = 100$ and $p = 1, 2$ or $p = 1, 2, 3$. The normally distributed random variables $N(0, 1/N)$ add the random component to $\widehat{\beta}_{Xj}$ that mimics the asymptotically normal distribution of the statistics obtained from GWAS with standardized data. The random error of $\widehat{\beta}_{Yjp}$, denoted by e_{Ypj}, is generated from a multivariate normal distribution for the multiple outcomes. All the variance terms of this multivariate normal distribution are set to $1/N$. When there are $P = 2$ outcomes, the covariance equals ρ/N with $\rho = 0$, 0.2, and 0.7 for the zero, low, and high outcome correlation settings respectively. When there are $P = 3$ outcomes, the pair-wise outcome correlation equals to $\rho_{ij} = \rho^{|i-j|}$ where $i, j \in \{1, 2, 3\}$ and $i \neq j$ with $\rho = 0$, 0.2 and 0.7 for three different settings. Then the standard errors of $\widehat{\beta}_{Xj}$ and $\widehat{\beta}_{Yjp}$ are given by

$$se(\widehat{\beta}_{Xj}) = se(\widehat{\beta}_{Yjp}) = \sqrt{1/N}.$$

To simulate the summary statistics, we need to set the values of the following parameters: θ_{kp} (the causal effect of the sub-component X_k on Y_p), δ_{kj} (the effect of variant G_j on X_k), η_j (the effect of variant G_j on the uncontrolled confounder U), and q_{xk}, q_{yp} (the effect of U on X_k and Y_p respectively). Let the variation in U explained by all the variants be h_U^2, and the variation in X_k directly explained by all the variants (not through U) be h_k^2. Then given the values of h_U^2 and h_k^2, parameters θ_{kp}, q_{xk} and q_{yp} are set as constants under the following restrictions to make $Var(X) = Var(Y_p) = 1$ feasible:

$$\sum_{k=1}^{K} h_k^2 + h_U^2 \sum_{k=1}^{K} q_{xk}^2 < 1, \qquad \sum_{k=1}^{K} \theta_{kp}^2 h_k^2 + h_U^2 \sum_{k=1}^{K} q_{xk}^2 \theta_{kp}^2 + q_y^2 h_U^2 < 1.$$

When there are $K = 4$ substantive clusters (with 15, 15, 30, 30 variants respectively) and one noise cluster (with 10 variants), let $h_U^2 = 0.05$ and $h_k^2 = (0, 0.05, 0.1, 0.1, 0.005)$ for $k = 1, ..., 5$ with the last entry for the junk cluster. Set $q_{xk} = 1$ for the first cluster, which corresponds to the correlated pleiotropy pathway, and $q_{xk} = 0$ for all the other clusters. When there are two outcomes, let $q_{y1} = 0.4$ and $q_{y2} = 0.1$. The causal effect parameters are set as $\theta_{k1} = (0.1, 0.3, 0.5, 0.4)$ for the first outcome, and $\theta_{k2} = (0.2, -0.3, 0.6, 0)$ for the second outcome, with $k = 1, ..., 4$. Causal effects of the 10 noise variants are generated from $N(0, 1)$. When there are three outcomes, additionally set $q_{y3} = 0.2$ and $\theta_{k3} = (-0.2, 0.3, 0, 0.3)$.

When there is $K = 1$ substantive cluster (with 90 variants) and one noise cluster (with 10 variants), set $h_U^2 = q_{xk} = q_{yp} = 0$ and $h_k^2 = (0.1, 0.005)$. The causal effects of X on all the outcomes are set to zero. In all simulation designs, δ_{kj} and η_j are generated from the uniform distribution $U[0.1, 0.3]$, and are randomly assigned to be positive or negative, then re-scaled as $\delta_{kj}\sqrt{h_k^2}/\sqrt{\sum_{j=1}^{J} \delta_{kj}^2}$ and $\eta_j\sqrt{h_U^2}/\sqrt{\sum_{j=1}^{J} \eta_j^2}$ to make sure that variations in U and X_k explained by the variants equal to h_u^2 and h_k^2 respectively.

D.2 Implementation of the methods

MR-AHC is performed with the outlier-robust variation as described in Section 2.3. To avoid spurious clusters, we only report the detected clusters containing more than 4 variants. Small clusters with less than 4 variants are subsumed into the junk cluster. An initial value of the proportion of noise variants is required for mclust and NAvMix, and we set this value favourably as 10% which is the ground truth. For these two methods, cluster membership is assigned based on the highest probability. The inputs for NAvMix are standardized before being supplied to the algorithm, as recommended in Grant et al. (2022). The cluster-specific causal estimates are obtained using the IVW approach. One exception is that if there is overdispersion within a detected cluster, as indicated by a non-zero I^2, then the cluster-specific estimate and its standard error are calculated using MR-RAPS (Zhao et al., 2020) to account for the within-cluster overdispersion.

D.3 Further simulation results

In the reported statistics, the Rand index (Rand, 1971) is a quantity which measures the similarity between two clustering outcomes with values between 0 and 1. It is given by $R = (a + b)/\binom{p}{2}$. Here, a denotes the number of pairs of objects that are classified as belonging to the same cluster in both clustering outcomes and b is the number of pairs of objects that are classified in different clusters by both clustering outcomes. R values close to 1 indicate good agreement and values close to 0 indicate poor agreement between two clustering outcomes. Here the Rand index is calculated with variants assigned to the substantive clusters by the methods. The mean absolute error (MAE) and mean squared error (MSE) are calculated as follows:

$$MAE_p = \frac{1}{J'} \sum_{j=1}^{J'} |\beta_{jk} - \widehat{\beta}_{jk}|, \quad MSE_p = \frac{1}{J'} \sum_{j=1}^{J'} (\beta_{jk} - \widehat{\beta}_{jk})^2,$$

where J' is the number of variants that are not assigned to the junk cluster by the methods, and do not belong to the junk cluster by the ground truth. β_{jk} is the true causal

effect associated with the cluster which G_j truly belongs to, and $\widehat{\beta}_{jk}$ is the causal estimate associated with the cluster which G_j is assigned to by the methods. The subscript p denotes the p-th outcome, and the overall MAE and MSE are calculated as means over all the outcomes.

We examine the original AHC method in the $P = 2$, $K = 4$ and $\rho = 0.7$ design. Since AHC requires individual-level data and does not have the option for multiple outcomes, we implement it in an equivalent way indirectly in the MR-AHC framework by setting the variances of the ratio estimates as 1 and covariances as 0 in the merging step. AHC shows a major drawback that it over-estimates the number of clusters, with 5.256 clusters averaging over 1000 simulation replications. MR-AHC which incorporates the variance-covariance matrix in the merging step achieves more accurate clustering results, with 4.153 clusters on average and better Rand index (0.950 for MR-AHC and 0.905 for AHC).

	MR-AHC	Mclust	Mclust noise	NavMix	NavMix ratio
**Three outcomes, k = 4, correlation = 0**					
# clusters	4.135	4.379	3.577	4.904	3.589
Rand index	0.942	0.892	0.865	0.773	0.861
# junk	14.086	0.000	9.996	4.122	5.064
# correct junk	9.404	0.000	9.169	3.138	4.351
MAE	0.084	0.101	0.102	0.112	0.100
MSE	0.029	0.036	0.036	0.040	0.038
**Three outcomes, k = 4, correlation = 0.2**					
# clusters	4.124	4.434	3.661	4.964	3.628
Rand index	0.944	0.892	0.869	0.776	0.862
# junk	14.310	0.000	10.098	4.726	5.060
# correct junk	9.490	0.000	9.229	3.409	4.304
MAE	0.084	0.101	0.101	0.111	0.099
MSE	0.029	0.036	0.035	0.039	0.037
**Three outcomes, k = 4, correlation = 0.7**					
# clusters	4.176	4.955	5.215	5.791	4.482
Rand index	0.958	0.944	0.960	0.798	0.904
# junk	14.899	0.000	9.377	6.807	4.079
# correct junk	9.516	0.000	8.586	4.557	3.329
MAE	0.083	0.090	0.084	0.104	0.091
MSE	0.028	0.032	0.029	0.037	0.033
**Three outcomes, k = 1, correlation = 0**					
# clusters	1.012	2.170	1.315	1.004	1.003
Rand index	0.996	0.901	0.920	0.818	0.818
# junk	13.884	0.000	11.108	0.130	0.127
# correct junk	9.829	0.000	9.519	0.013	0.019
MAE	0.011	0.020	0.015	0.016	0.017
MSE	0.000	0.003	0.001	0.000	0.000
Freq.null	0.975	0.815	0.758	0.998	0.993
**Three outcomes, k = 1, correlation = 0.2**					
# clusters	1.006	2.226	1.367	1.015	1.009
Rand index	0.997	0.888	0.906	0.814	0.816
# junk	13.911	0.000	11.083	0.594	0.627
# correct junk	9.896	0.000	9.500	0.076	0.072
MAE	0.011	0.021	0.016	0.016	0.017
MSE	0.000	0.003	0.001	0.000	0.001
Freq.null	0.974	0.784	0.735	0.997	0.983
**Three outcomes, k = 1, correlation = 0.7**					
# clusters	1.008	2.180	1.297	1.932	1.935
Rand index	0.995	0.916	0.941	0.535	0.537
# junk	13.514	0.000	10.935	48.183	48.256
# correct junk	9.940	0.000	9.687	8.038	8.096
MAE	0.012	0.019	0.014	0.030	0.132
MSE	0.000	0.002	0.001	0.002	0.019
Freq.null	0.952	0.801	0.764	0.869	0.002

Table S1: Simulation results for designs with three outcomes. All methods are conducted treating the outcome correlations as 0. Statistics are calculated as the mean over 1000 replications.

	MR-AHC	Mclust	Mclust noise	NavMix	NavMix ratio
**Two outcomes, k = 4, correlation = 0**					
# clusters	4.196	3.715	2.963	3.018	2.307
Rand index	0.917	0.757	0.737	0.615	0.710
# junk variants	10.966	0.000	7.726	6.517	4.080
# correct junk	6.975	0.000	7.135	1.446	3.951
MAE	0.088	0.120	0.123	0.162	0.106
MSE	0.030	0.041	0.041	0.052	0.035
**Two outcomes, k = 4, correlation = 0.2**					
# clusters	4.163	3.665	2.933	3.074	2.161
Rand index	0.919	0.744	0.725	0.620	0.687
# junk variants	11.268	0.000	7.833	1.281	4.083
# correct junk	7.257	0.000	7.233	0.315	4.007
MAE	0.088	0.118	0.122	0.156	0.101
MSE	0.030	0.039	0.040	0.049	0.032
**Two outcomes, k = 4, correlation = 0.7**					
# clusters	4.153	3.652	3.754	3.784	2.519
Rand index	0.950	0.784	0.835	0.684	0.751
# junk variants	12.992	0.000	8.044	4.907	4.994
# correct junk	8.271	0.000	7.433	0.909	4.648
MAE	0.083	0.091	0.088	0.113	0.096
MSE	0.028	0.027	0.027	0.031	0.026
**Two outcomes, k = 1, correlation = 0**					
# clusters	1.026	2.211	1.312	1.000	1.001
Rand index	0.947	0.887	0.895	0.818	0.818
# junk variants	10.194	0.000	8.211	0.000	0.000
# correct junk	7.703	0.000	7.073	0.000	0.000
MAE	0.012	0.020	0.015	0.017	0.017
MSE	0.000	0.004	0.001	0.000	0.000
Freq.null	0.955	0.726	0.767	0.998	0.997
**Two outcomes, k = 1, correlation = 0.2**					
# clusters	1.039	2.226	1.297	1.001	1.001
Rand index	0.948	0.881	0.893	0.818	0.818
# junk variants	10.563	0.000	8.438	0.015	0.000
# correct junk	7.919	0.000	7.199	0.001	0.000
MAE	0.012	0.021	0.015	0.017	0.017
MSE	0.000	0.004	0.001	0.000	0.000
Freq.null	0.950	0.690	0.794	0.997	0.996
**Two outcomes, k = 1, correlation = 0.7**					
# clusters	1.008	2.221	1.296	1.002	1.001
Rand index	0.978	0.910	0.924	0.818	0.818
# junk variants	12.155	0.000	9.458	0.017	0.017
# correct junk	9.122	0.000	8.323	0.001	0.003
MAE	0.011	0.019	0.014	0.017	0.017
MSE	0.000	0.003	0.001	0.000	0.000
Freq.null	0.979	0.413	0.778	0.935	0.935

Table S2: Simulation results for designs with two outcomes. All methods are conducted with the true correlation parameters where feasible. Statistics are calculated as the mean over 1000 replications.

	MR-AHC	Mclust	Mclust noise	NavMix	NavMix ratio
**Three outcomes, k = 4, correlation = 0**					
# clusters	4.046	4.379	3.577	4.907	3.589
Rand index	0.948	0.892	0.865	0.773	0.861
# junk	19.616	0.000	9.996	4.122	5.064
# correct junk	9.579	0.000	9.169	3.140	4.351
MAE	0.084	0.101	0.102	0.112	0.100
MSE	0.029	0.036	0.036	0.040	0.038
**Three outcomes, k = 4, correlation = 0.2**					
# clusters	4.110	4.434	3.659	4.878	3.546
Rand index	0.948	0.892	0.869	0.774	0.866
# junk	14.385	0.000	10.099	4.533	5.406
# correct junk	9.575	0.000	9.228	3.287	4.561
MAE	0.084	0.101	0.101	0.112	0.098
MSE	0.029	0.036	0.035	0.039	0.037
**Three outcomes, k = 4, correlation = 0.7**					
# clusters	4.215	4.955	5.215	6.033	3.583
Rand index	0.981	0.944	0.960	0.804	0.898
# junk	16.019	0.000	9.377	5.885	6.328
# correct junk	9.819	0.000	8.586	4.255	5.463
MAE	0.081	0.090	0.084	0.100	0.093
MSE	0.027	0.032	0.029	0.030	0.030
**Three outcomes, k = 1, correlation = 0**					
# clusters	1.004	2.170	1.315	1.004	1.003
Rand index	0.997	0.901	0.920	0.818	0.818
# junk	18.908	0.000	11.108	0.130	0.127
# correct junk	9.877	0.000	9.519	0.013	0.019
MAE	0.012	0.020	0.015	0.016	0.017
MSE	0.000	0.003	0.001	0.000	0.000
Freq.null	0.978	0.815	0.758	0.998	0.993
**Three outcomes, k = 1, correlation = 0.2**					
# clusters	1.002	2.226	1.368	1.003	1.001
Rand index	0.998	0.887	0.906	0.817	0.818
# junk	14.044	0.000	11.080	0.417	0.011
# correct junk	9.924	0.000	9.499	0.037	0.001
MAE	0.011	0.021	0.016	0.016	0.016
MSE	0.000	0.003	0.001	0.000	0.000
Freq.null	0.979	0.789	0.739	0.999	0.997
**Three outcomes, k = 1, correlation = 0.7**					
# clusters	1.000	2.180	1.298	1.002	1.004
Rand index	1.000	0.916	0.941	0.817	0.818
# junk	14.205	0.000	10.936	0.409	0.098
# correct junk	9.999	0.000	9.686	0.024	0.012
MAE	0.011	0.019	0.014	0.016	0.016
MSE	0.000	0.002	0.001	0.000	0.000
Freq.null	0.958	0.522	0.771	0.951	0.947

Table S3: Simulation results for designs with three outcomes. All methods are conducted with the true correlation parameters where feasible. Statistics are calculated as the mean over 1000 replications.

	$K = 4$		$K = 1$	
	MR-AHC	MR-Clust	MR-AHC	MR-Clust
#clusters	3.961	4.119	1.212	1.150
Rand index	0.920	0.918	0.919	0.898
#junk	6.018	2.548	8.484	4.667
# correctjunk	3.313	2.441	5.981	4.644
MAE	0.089	0.085	0.017	0.011
MSE	0.029	0.028	0.002	0.000

Table S4: Simulation results for designs with one outcome using MR-AHC and MR-Clust(Foley et al., 2021). Data are simulated similarly as in the main text. When there are $K = 4$ substantive clusters, set $\beta = (0.1, 0.3, -0.5, 0)$ and $q_y = 0.4$. With $K = 1$, set $\beta = 0$. All other parameters are the same as in the main text. Statistics are calculated as the mean over 1000 replications.

E Extended simulation studies

We conduct further simulation studies as an extension to the main simulations presented in Section 3 and Appendix D. In this section, we present simulation results and related discussions concerning the post-clustering inference, performance of the methods with alternative pleiotropy setup and weak instrumental variables.

E.1 Post-clustering inference

We report the coverage rates of the 95% confidence intervals (CI) of the post-clustering estimates generated from the main simulations presented in Section 3 and Appendix D. First of all, for each detected cluster, we need to decide which true cluster it is referring to, so that we can calculate the CI coverage rate using the cluster-specific estimates and the corresponding true causal parameters. We propose a matching process based on the Jaccard index, which measures the similarity between two clusters. The Jaccard index is given by $J = \frac{|A \cap B|}{|A \cup B|}$, which is the ratio of the number of elements in the intersection of the

two sets A and B and the number of elements in the union of the two sets. The value of the Jaccard index is between 0 and 1. A value close to 1 indicates that two clusters are similar.

We illustrate the matching process using the following hypothetical examples. Suppose we have three true variant clusters, and a particular clustering approach detects four variant clusters. We then calculate the Jaccard index between each true cluster and each detected cluster, forming a Jaccard matrix presented in Table S5.

	True cluster 1	True cluster 2	True cluster 3
Detected cluster 1	**0.9**	0.8	0.3
Detected cluster 2	0.7	**0.5**	0.1
Detected cluster 3	0.2	0.2	**0.75**
Detected cluster 4	0	0.1	0

Table S5: Matching true clusters to detected clusters using the Jaccard matrix with hypothetical data. Example 1.

First we find the true-detected cluster pair with the highest Jaccard index, which in this case is true 1-detected 1 with a Jaccard index of 0.9. This means that we match true cluster 1 with detected cluster 1. We then remove these two clusters from the matching process, and find the the true-detected pair with the highest Jaccard index amongst the remaining clusters, which is true cluster 3 and detected cluster 3 with 0.75, making the second match. Remove them from the matching process and repeat the steps for the remaining clusters, we then find the third pair which is true cluster 2-detected cluster 2. In this way we find a match for all three true clusters.

It might also be the case that there are more true clusters than detected clusters, for example, the Jaccard matrix presented in Table S6. In this case, we need to re-use one of the detected clusters to have a match for every true cluster. We adjust the aforementioned matching process in the following way: again we start with the true-detected cluster pair with the highest Jaccard index which is true 1-detected 1, but instead of

removing both of them from the matching process, here we keep detected cluster 1 and only remove true cluster 1. Next we find the pair with the highest Jaccard index amongst the remaining clusters, which results in true 2-detected 1. In this case we assign detected cluster 1 to true cluster 2 as well so it is the detected cluster we need to re-use. After we find this re-used cluster, we remove all the true and detected clusters that have found a match from the process so that the matching process is only amongst true cluster 3-4 and detected cluster 2-3. Next the pair with the highest Jaccard index is true 3-detected 3 with 0.75 and we remove both of them from the process. Repeat the steps and true cluster 4 is assigned to detected cluster 2.

	True cluster 1	True cluster 2	True cluster 3	True cluster 4
Detected cluster	**0.9**	**0.8**	0.3	0.1
Detected cluster	0.7	0.5	0.1	**0.5**
Detected cluster	0.2	0.2	**0.75**	0.6

Table S6: Matching true clusters to detected clusters using the Jaccard matrix with hypothetical data. Example 2.

After this matching process, we calculate the total coverage of the estimated 95% CIs in the following manner: for each true-detected cluster pair, since there can be multiple outcomes hence multiple true causal effects and causal estimates, we check if all of the estimated CIs contain the true causal parameters, assigning a value of 1 if they do and 0 otherwise. We then report the average CI coverage across all the true-detected cluster pairs. The post-clustering cluster-specific estimates and their standard errors are calculated with the IVW approach. For clusters with within-cluster overdispersion indicated by $I^2 > 0$, we report the MR-RAPS estimates and standard errors correcting for overdispersion. For scenarios with $K = 4$, we calculate the CI coverage for the three non-pleiotropic clusters.

The simulation results are presented in Table S7. Overall, MR-AHC has the highest CI coverage among all the methods with $K = 4$. It also has the most stable coverage rates across all settings. Its coverage rates are higher with $K = 1$ than those with $K = 4$.

However, the CI coverage is in generally lower than the nominal level 0.95, which is in line with the well-established theoretical results in post-selection inference that consistent selection procedures still lead to under-covered CIs (Leeb and Pötscher, 2005; Patel et al., 2021). But it should be noted that these coverage rates incorporate both the bias stemmed from the clustering process and the matching procedure described above.

	MR-AHC	mclust	mclust noise	NAvMix	NAvMix ratio
**Two outcomes, K = 4**					
$\rho = 0$	0.767	0.498	0.412	0.351	0.470
$\rho = 0.2$	0.769	0.515	0.421	0.395	0.508
$\rho = 0.7$	0.777	0.687	0.716	0.532	0.596
**Two outcomes, K = 1**					
$\rho = 0$	0.874	0.877	0.865	0.988	0.988
$\rho = 0.2$	0.865	0.863	0.866	0.989	0.985
$\rho = 0.7$	0.885	0.887	0.881	0.961	0.143
**Three outcomes, K = 4**					
$\rho = 0$	0.732	0.490	0.423	0.486	0.580
$\rho = 0.2$	0.743	0.495	0.436	0.492	0.594
$\rho = 0.7$	0.775	0.708	0.785	0.657	0.764
**Three outcomes, K = 1**					
$\rho = 0$	0.817	0.818	0.812	0.980	0.979
$\rho = 0.2$	0.815	0.798	0.804	0.976	0.970
$\rho = 0.7$	0.843	0.845	0.862	0.946	0.011

Table S7: Coverage rates of the 95% confidence intervals of the post-clustering cluster-specific estimates. The causal estimates and standard errors for each cluster are calculated with the inverse-variance weighted (IVW) approach. For clusters with within-cluster overdispersion indicated by $I^2 > 0$, we report the MR-RAPS estimates and standard errors correcting for overdispersion. Detected clusters are matched to the true clusters based on the matching process described in the text. The confidence interval converge is an average across all pairs of detected and true cluster. For scenarios with $K = 4$, we calculate confidence interval coverage for the three non-pleiotropic clusters. "*mclust noise*" stands for the mclust algorithm with a noise component, and "*NAvMix ratio*" for the NAvMix method with ratio estimates as input. Coverage rates are calculated as the mean over 1000 replications.

In an attempt to improve the CI coverage, we have searched the literature aiming for post-selection/clustering inference methods that do not require individual-level data. We found several such papers specifically fit in the summary-date MR framework. For example, Bi et al. (2019) introduce a conditional selective inference approach that accounts for the bias originated from a certain class of IV selection procedures. Patel et al. (2021) develop a IV

selection method and subsequently propose a post-selection inference procedure for that. However, these methods are not feasible for MR-AHC, as their focus is valid IV selection and assume a homogeneous causal effect, which is quite different setup from MR-AHC which allows for heterogeneous causal effects and inherently does not classify variants as valid of invalid IV. Also, these inference approaches are mainly developed for specific IV selection procedures thus can be difficult to apply to other methods directly. Research on post-selection inference in the field of econometrics is generally based on individual-level data thus not feasible for MR-AHC. But some methods can be translated to the summary-data setup directly, for example, the searching method proposed by Guo (2023). However, this method suffers has same problem that it is designed for IV selection with a homogeneous causal effect and relies on certain assumptions on the number of valid instruments which are not valid for MR-AHC. Therefore, we leave as future research the topic of post-clustering (rather than post-selection) inference allowing for GWAS summary statistics.

E.2 Alternative pleiotropy setup

In this section we provide simulation results and discussion about how different pleiotropy settings can affect cluster identification. In the main simulations presented in Section 3 and Appendix D, we examine the performance of the methods under the model setup with only correlated pleiotropy and no direct effects from the pleiotropic variants to the exposure, thus pleiotropic variants have the same ratio estimand. Here we extend the previous simulations and set the pleiotropic variants to have direct effects on the corresponding exposure sub-component. In this case, pleiotropic variants have different ratio estimands thus in principle do not belong to the same cluster, although they affect the phenotypes through the same biological pathway (see the discussion in Appendix C.1).

We use the same parameter specification as in the main simulations with the following exception. When $K = 4$, we make the 15 variants in Cluster 1 (i.e. the pleiotropic

variants) have direct effects $\boldsymbol{\delta_1}$ generated from a uniform distribution $U[0.1, 0.3]$, then randomly assigned as positive or negative and scaled so that these variants jointly explain 5% variation in X (i.e. the first entry of h_k^2 becomes 0.05 instead of 0). When $K = 1$, we generate $\boldsymbol{\eta}$ from a uniform distribution $U[0.1, 0.3]$, then randomly assigned as positive or negative and scaled so the that 90 variants in Cluster 1 jointly explain 5% variation in U (i.e. h_u^2 becomes 0.05 instead of 0). We focus on the scenarios with $\rho = 0$. The model setup and representative simulated data scatter plots (with two outcomes) are illustrated in Figure S3.

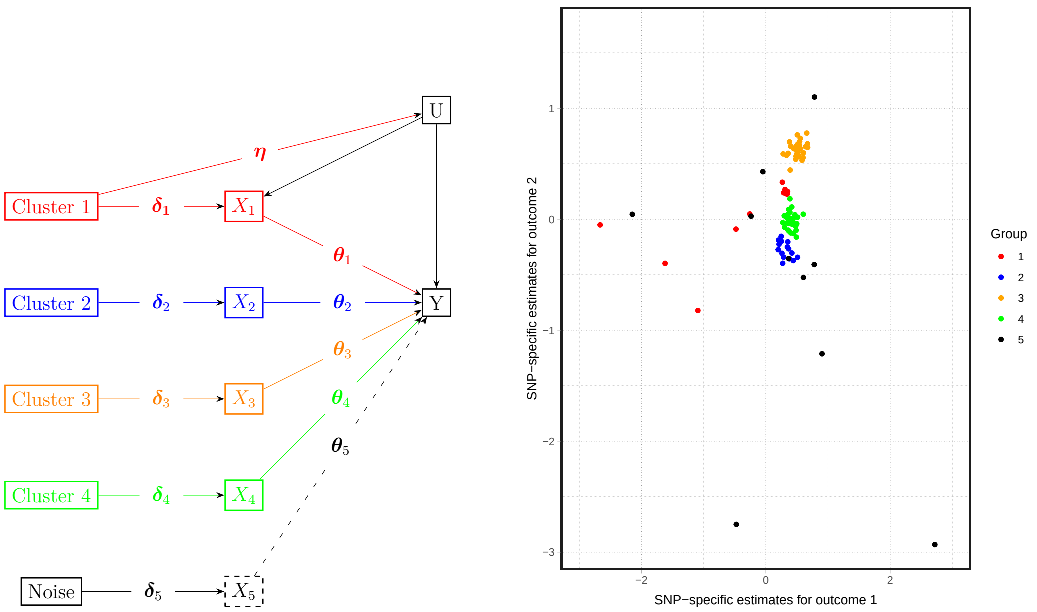

(a) Simulation designs with 4 substantive variant clusters.

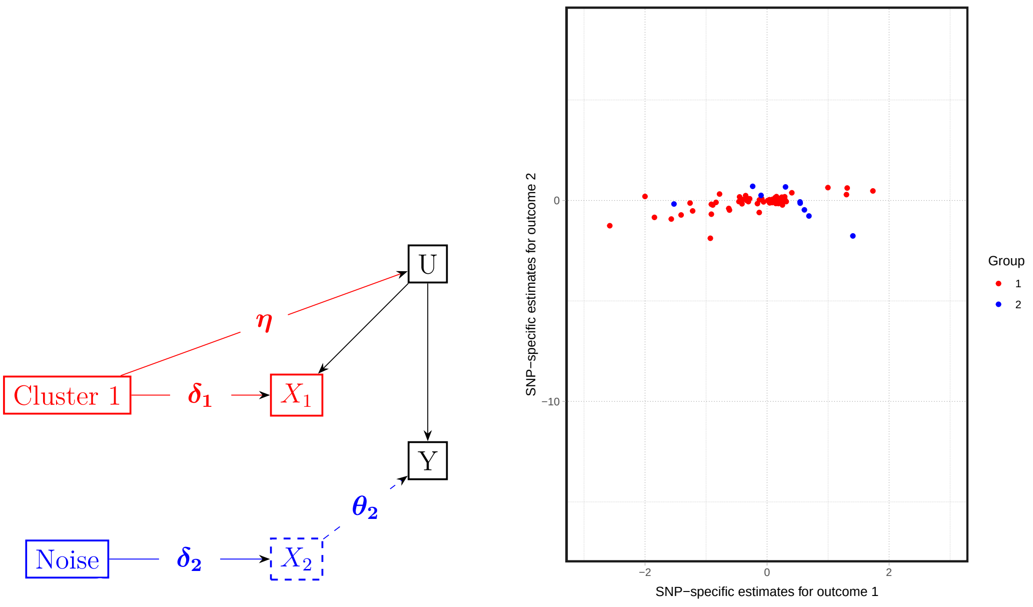

(b) Simulation designs with 1 substantive variant cluster.

Figure S3: (Left in each panel) Directed acyclic graphs (DAG) of the data generation process and (right in each panel) scatter plots of representative simulated data with two outcomes. In all settings we set $\rho = 0$. In the scatter plots, on the x-axis are the ratio estimates for the first outcome, and on the y-axis for the second outcome. Each point represents a specific variant.

We report the simulation results in Table S8. The new statistic "*Rand index (pleiotropy)*" is the Rand index only for the 15 variants in Cluster 1. All the other statistics are defined in the same way as in the main simulations. Although by definition the variants in

Cluster 1 may not belong to the same cluster, we still treat them as in the same cluster when calculating the overall Rand index and the junk variants related results, as they do belong to the same biological pathway.

As expected, "*Rand index (pleiotropy)*" is much lower than 1 for all methods in all settings, as these pleiotropic variants essentially do not have the same ratio estimands and may fall into the junk variants category. In line with this, MR-AHC selects even more variants as junk than in the main simulations. It also tends to detect more clusters than the ground truth, as the pleiotropic variants are frequently split into different clusters. When $K = 4$, the overall Rand index seems to improve in comparison with the main simulations, which is probably because it is calculated with variants selected as non-junk thus more variants selected as junk may be favourable for that.

While with $K = 4$ MR-AHC still performs reasonably well and outperforms all other approaches, a major breakdown arises with $K = 1$. All methods tend to over-estimate the number of clusters than the ground truth of 1, but it is particularly pronounced for MR-AHC. This also results in a low Rand index, both overall and for the pleiotropic variants. These results are not entirely unanticipated. In this setting, all 90 variants in Cluster 1 have variant-specific ratio estimands, thus all 100 variants (there are 10 variants in the noise cluster) essentially may not form any cluster. This represents an extreme case where all variants involved in the analysis are 'noise' and there are no real clusters.

With $K = 1$, we re-run the simulations by setting $\eta = 0$ for some variants so that they have the same ratio estimand and do form a cluster. We vary the number of such variants and the statistic "*%non-noise*" in Table S9 represents the percentage of these variants among all 100 variants. We can see clearly that the performance of MR-AHC improves as the non-noise percentage increases. But more outcomes requires larger non-noise percentage for the method to detect less spurious clusters and achieve higher Rand indices, which is reflected by the results with two outcomes versus with three outcomes.

	MR-AHC	mclust	mclust noise	NAvMix	NAvMix ratio
**Two outcomes, K = 4**					
# clusters	4.266	4.324	3.604	3.719	2.319
Rand index	0.941	0.800	0.828	0.685	0.738
Rand index (*pleiotropy*)	0.484	0.436	0.417	0.321	0.372
# junk variants	19.820	0.000	9.065	3.493	7.823
# correct junk	7.020	0.000	4.620	0.869	4.149
**Three outcomes, K = 4**					
# clusters	4.059	4.667	4.408	4.784	3.770
Rand index	0.964	0.875	0.907	0.761	0.860
Rand index (*pleiotropy*)	0.531	0.440	0.409	0.239	0.396
# junk variants	21.762	0.000	10.842	4.890	3.538
# correct junk	9.303	0.000	6.066	3.373	2.253
**Two outcomes, K = 1**					
# clusters	3.655	3.901	2.531	1.998	1.908
Rand index	0.447	0.486	0.504	0.502	0.566
Rand index (*pleiotropy*)	0.332	0.443	0.417	0.413	0.449
# junk variants	18.207	0.000	6.686	15.381	17.889
# correct junk	6.204	0.000	0.977	4.617	4.784
**Three outcomes, K = 1**					
# clusters	3.879	3.972	2.925	2.004	1.993
Rand index	0.434	0.469	0.498	0.500	0.531
Rand index (*pleiotropy*)	0.334	0.399	0.387	0.421	0.446
# junk variants	18.652	0.000	6.976	13.160	13.642
# correct junk	8.125	0.000	0.894	4.213	4.426

Table S8: Simulation results for alternative pleiotropy setup. In all settings we set $\rho = 0$. We perform MR-AHC with the iterated outlier removal procedure for $K = 1$. "*mclust noise*" stands for the mclust algorithm with a noise component, and "*NAvMix ratio*" for the NAvMix method with ratio estimates as input. Statistics are calculated as the mean over 1000 replications.

	%non-noise = 0.3	%non-noise = 0.5	%non-noise = 0.6	%non-noise = 0.7
**Two outcomes, K = 1**				
# clusters (*mean*)	3.199	2.423	1.954	1.397
Rand index (*mean*)	0.481	0.592	0.705	0.854
# clusters (*median*)	3.000	2.000	2.000	1.000
Rand index (*median*)	0.461	0.516	0.640	0.931
**Three outcomes, K = 1**				
# clusters (*mean*)	3.423	2.645	2.276	2.036
Rand index (*mean*)	0.458	0.540	0.602	0.635
# clusters (*median*)	3.000	3.000	2.000	2.000
Rand index (*median*)	0.437	0.506	0.542	0.565

Table S9: Simulation results for MR-AHC with varying percentage of variants forming a cluster for $K = 1$. In all settings we set $\rho = 0$. We perform MR-AHC with the iterated outlier removal procedure. Statistics are calculated over 1000 replications.

E.3 Weak instrumental variables

In this section, we examine the performance of the clustering methods with varying instrument strength. We focus on the settings with two outcomes and set $\rho = 0$. In the main simulations presented in Sections 3 and Appendix D, instrument strength is controlled by two parameters h_k^2 (the proportion of variation in X_k explained by the variants) and h_U^2 (the proportion of variation in U explained by the variants). We control for the overall strength of the instruments by a factor $\boldsymbol{C_{\mathcal{F}}}$. First with $K = 4$, we set $\boldsymbol{C_{\mathcal{F}}} = 0.32$ and generate data with $\boldsymbol{C_{\mathcal{F}}} \times h_k^2$ and $\boldsymbol{C_{\mathcal{F}}} \times h_U^2$. This results in an overall instrument strength measured by the F statistic with $F = 53.04$. We choose this value because it mimics the instrument strength of our real-world application example. We then further decrease the instrument strength by setting $\boldsymbol{C_{\mathcal{F}}} = 0.1$ with $F = 16.60$ and eventually a weak IV setup with $\boldsymbol{C_{\mathcal{F}}} = 0.05$ and $F = 8.73$ which falls below the conventional rule of thumb cut-off value of 10 (Staiger and Stock, 1997). For the $K = 1$ setup we set the values of $\boldsymbol{C_{\mathcal{F}}}$ accordingly so the F statistics match with those in the $K = 4$ settings.

The simulation results on the number of detected clusters and the Rand index are presented in Table S10 for $K = 4$ and Table S11 for $K = 1$. With $K = 4$, MR-AHC outperforms all other approaches across all settings. It still achieves clustering results that are close to the ground truth with $F = 53.04$. However, with weaker instruments, it tends to under-estimate the number of clusters and the Rand index decreases accordingly. This pattern is also observed for all other approaches. These results are to some extent expected when observing the scatter plot of a representative simulated dataset with $F = 8.73$ in Figure S4. All clusters diffuse into each other, thus it can be difficult for the methods to distinguish and identify the clusters. When $K = 1$, all methods perform better in comparison with $K = 4$. Again, MR-AHC has the best performance among all methods and performs reasonably well even in the weak IV setup.

	MR-AHC	mclust	mclust noise	NAvMix	NAvMix ratio
**Two outcomes, K = 4, $C_{\mathcal{F}} = 0.32$, $F = 53.04$**					
# clusters (*mean*)	4.186	4.213	2.875	2.045	1.356
Rand index (*mean*)	0.854	0.785	0.730	0.504	0.353
# clusters (*median*)	4.000	4.000	3.000	2.000	1.000
Rand index (*median*)	0.855	0.813	0.791	0.501	0.240
**Two outcomes, K = 4, $C_{\mathcal{F}} = 0.1$, $F = 16.60$**					
# clusters (*mean*)	3.546	3.691	2.143	1.630	1.027
Rand index (*mean*)	0.714	0.563	0.518	0.402	0.255
# clusters (*median*)	3.000	3.000	2.000	2.000	1.000
Rand index (*median*)	0.715	0.555	0.523	0.493	0.245
**Two outcomes, K = 4, $C_{\mathcal{F}} = 0.05$, $F = 8.73$**					
# clusters (*mean*)	3.214	3.599	2.148	1.393	1.006
Rand index (*mean*)	0.634	0.543	0.508	0.334	0.263
# clusters (*median*)	3.000	3.000	2.000	1.000	1.000
Rand index (*median*)	0.644	0.549	0.516	0.227	0.258

Table S10: Simulations with varying instrumental variable strength for settings with two outcomes and $K = 4$. In all settings we set $\rho = 0$. "*mclust noise*" stands for the mclust algorithm with a noise component, and "*NAvMix ratio*" for the NAvMix method with ratio estimates as input. Statistics are calculated over 1000 replications.

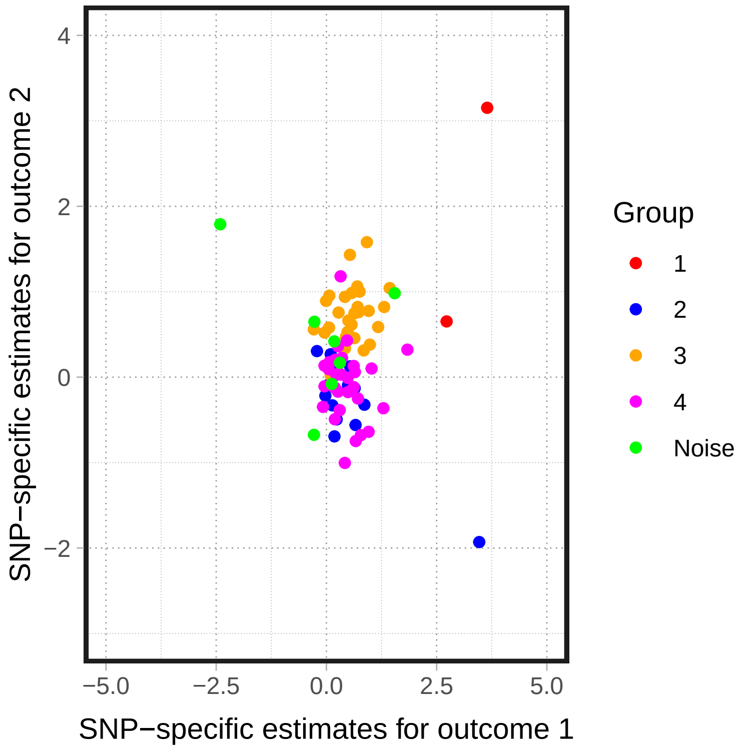

Figure S4: A scatter plot of representative simulated data for $K = 4$ and $F = 8.73$.

We have looked into the clustering of MR-AHC with $K = 4$ in the weak IV setup. In the merging step, the Wald test requires asymptotic normal distribution of the cluster-specific IVW estimator, which is asymptotically equivalent to the Two-Stage Least Squares (2SLS) estimator with continuous outcomes (Burgess et al., 2019). In the downward testing step, the Cochran's Q statistic is identical to the Sargan overidenfication testing statistic (Bowden and Holmes, 2019). According to the results in Staiger and Stock (1997) under weak instrument asymptotics, the 2SLS estimator is inconsistent and non-normal, and the testing statistics for overidenfication restrictions has non-standard asymptotic distributions. Therefore, both the merging step and the downward testing step are affected by weak instruments. For example, we observed in the simulations the merging step of MR-AHC and found that variants belonging to different true clusters merge into one cluster incorrectly at very early stage of the merging step. Also, the Q test fails to reject clusters formed by variants from mixed true clusters. Both the Wald test and Q test are under-powered in the presence of weak instruments.

	MR-AHC	mclust	mclust noise	NAvMix	NAvMix ratio
**Two outcomes, K = 1, $C_{\mathcal{F}} = 0.8$, $F = 51.39$**					
# clusters (*mean*)	1.044	2.238	1.299	1.002	1.000
Rand index (*mean*)	0.934	0.876	0.892	0.818	0.818
# clusters (*median*)	1.000	2.000	1.000	1.000	1.000
Rand index (*median*)	0.936	0.904	0.936	0.818	0.818
**Two outcomes, K = 1, $C_{\mathcal{F}} = 0.25$, $F = 16.75$**					
# clusters (*mean*)	1.048	2.799	1.435	1.003	1.001
Rand index (*mean*)	0.891	0.785	0.833	0.818	0.818
# clusters (*median*)	1.000	2.000	1.000	1.000	1.000
Rand index (*median*)	0.897	0.802	0.880	0.818	0.818
**Two outcomes, K = 1, $C_{\mathcal{F}} = 0.12$, $F = 8.56$**					
# clusters (*mean*)	1.043	3.246	1.719	1.002	1.003
Rand index (*mean*)	0.855	0.709	0.750	0.818	0.818
# clusters (*median*)	1.000	3.000	2.000	1.000	1.000
Rand index (*median*)	0.863	0.727	0.745	0.818	0.818

Table S11: Simulations with varying instrumental variable strength for settings with two outcomes and $K = 1$. In all settings we set $\rho = 0$. "*mclust noise*" stands for the mclust algorithm with a noise component, and "*NAvMix ratio*" for the NAvMix method with ratio estimates as input. Statistics are calculated over 1000 replications.

A straightforward potential solution is to replace the IVW estimate and the Q statistic by a weak IV robust counterpart. For example, the 'exact' IVW estimate proposed by Bowden et al. (2019), which is an equivalence of the Limited information maximum likelihood (LIML) estimator in the two-sample summary-data setup. A modified Q statistic can then be obtained with this exact IVW estimate and is also robust to weak instruments (Wang and Kang, 2022). However, the adjusted algorithm with the replacement does not improve the performance of the method with overall weak instruments. When looking into the merging results with exact IVW estimates, variants from different true clusters still merge incorrectly at early stage.

Therefore, the weak instrument robust methods that treat all instruments collectively might not be suitable for MR-AHC, as the method always begins by dealing with individual instruments, thus may require consideration of the strength of each individual instrument. Now we consider a setup where only part of the variants are weak. In the setting with $K = 4$, we take 5 variants from each of the four clusters and make them weak instruments. We change the values of δ_j (the direct effect from the variant to the exposure) and η_j (the direct effect from the variant to U) of these variants so that their average strength, as measured by the F statistic, is $F_w = 7.42$, $F_w = 1.71$ and $F_w = 1.01$ respectively in three settings. In the last scenario with $F_w = 1.01$, these variants are essentially irrelevant instruments. We keep the strength of all the other variants the same as in the main simulations. For $K = 1$, we make 10 of the variants weak in a similar way with $F_w = 5.91$, $F_w = 1.25$ and $F_w = 1.01$.

To deal with the individual weak instruments, Guo et al. (2018) propose a first-stage screening procedure in their IV selection method, operates as follows: for each variant G_j, conduct a t-test for the null hypothesis of $\gamma_j = 0$ (γ_j represents the overall effect of G_j on X). Rule out G_j from the subsequent clustering process if the test statistic t_j is smaller than a cut-off value, which is defined as $\sqrt{2.01 \times log(n)}$ with n being the sample size. We conduct simulations for MR-AHC with and without the first-stage screening in

the some weak instruments setup described above. The results are presented in Table S12 for $K = 4$ and Table S13 for $K = 1$. The statistic "*# weak*" is the number of variants selected as weak by the first-stage screening. The true value is 20 for $K = 4$ and 10 for $K = 1$. "*# correct weak*" is the number of true weak instruments selected as weak by the procedure.

The first-stage screening performs well in detecting all the true weak instruments. It helps improve the performance of MR-AHC, especially in the $K = 4$ settings with very weak instruments in terms of detecting the correct number of clusters. It should be noted that if all the variants involved in the analysis are genome-wide significant ($p < 5 \times 10^{-8}$, as in our real-world application example), then the first-stage screening may not be necessary as the t-statistics are likely to exceed the cut-off value $\sqrt{2.01 \times log(n)}$.

To summarize, MR-AHC tends to under-estimate the number of clusters when the overall IV strength is weak. But it should not be a significant concern if all the involved variants are genome-wide significant, which is common in MR studies. If some of the genetic instruments are weak, the first-stage screening procedure by Guo et al. (2018) may be beneficial, although further investigation of the performance of the procedure in various clustering settings and how it affects the post-clustering inference might be warranted.

	MR-AHC	MR-AHC (*first-stage*)
**Two outcomes, K = 4, $F_w = 7.42$**		
# weak (*mean*)	NA	23.33
# correct weak (*mean*)	NA	19.38
# clusters (*mean*)	4.273	4.124
Rand index (*mean*)	0.850	0.917
# clusters (*median*)	4.000	4.000
Rand index (*median*)	0.851	0.920
**Two outcomes, K = 4, $F_w = 1.71$**		
# weak (*mean*)	NA	23.96
# correct weak (*mean*)	NA	20.00
# clusters (*mean*)	4.675	4.121
Rand index (*mean*)	0.840	0.920
# clusters (*median*)	5.000	4.000
Rand index (*median*)	0.843	0.924
**Two outcomes, K = 4, $F_w = 1.01$**		
# weak (*mean*)	NA	23.96
# correct weak (*mean*)	NA	20.00
# clusters (*mean*)	4.997	4.121
Rand index (*mean*)	0.839	0.920
# clusters (*median*)	5.000	4.000
Rand index (*median*)	0.841	0.924

Table S12: Simulation results for MR-AHC with some weak instrumental variables for settings with two outcomes and $K = 4$. In all settings we set $\rho = 0$. Statistics are calculated over 1000 replications.

	MR-AHC	MR-AHC (*first-stage*)
**Two outcomes, K = 1, $F_w = 5.91$**		
# weak (*mean*)	NA	22.84
# correct weak (*mean*)	NA	9.86
# clusters (*mean*)	1.118	1.013
Rand index (*mean*)	0.931	0.968
# clusters (*median*)	1.000	1.000
Rand index (*median*)	0.936	0.971
**Two outcomes, K = 1, $F_w = 1.25$**		
# weak (*mean*)	NA	22.98
# correct weak (*mean*)	NA	10.00
# clusters (*mean*)	1.253	1.012
Rand index (*mean*)	0.911	0.969
# clusters (*median*)	1.000	1.000
Rand index (*median*)	0.933	0.971
**Two outcomes, K = 1, $F_w = 1.01$**		
# weak (*mean*)	NA	22.98
# correct weak (*mean*)	NA	10.00
# clusters (*mean*)	1.282	1.012
Rand index (*mean*)	0.907	0.969
# clusters (*median*)	1.000	1.000
Rand index (*median*)	0.933	0.971

Table S13: Simulation results for MR-AHC with some weak instrumental variables for settings with two outcomes and $K = 1$. In all settings we set $\rho = 0$. Statistics are calculated over 1000 replications.

F Further application details

F.1 Post-clustering estimation results

Cluster	# SNPs	Est-T2D	t-T2D	Est-OA	t-OA	Q p-value	I^2
1	124	1.326<br>(0.073)	18.236	1.250<br>(0.054)	23.321	0.929	0.000
2	258	1.076<br>(0.051)	21.102	0.067<br>(0.037)	1.791	0.951	0.000
3	32	−1.068<br>(0.138)	7.718	0.738<br>(0.101)	7.327	1.000	0.000
4	22	−2.799<br>(0.190)	14.704	0.103<br>(0.143)	0.722	0.437	0.019

Table S14: The estimation results using SNPs in each cluster detected by MR-AHC for the causal relationship between BFP and T2D-OA. We report the number of SNPs in the cluster (*#SNPs*), the cluster-specific inverse-variance weighted (IVW) estimate (*Est*), the standard error (in the parentheses under the estimates) and associated t-statistic (t), and statistics measuring the heterogeneity within each cluster (p-value of the Q statistic and the I^2 statistic). For Cluster 4 with within-cluster overdispersion indicated by $I^2 > 0$, we report the MR-RAPS estimates and standard errors correcting for overdispersion.

F.2 Post-clustering pathway analysis

We map SNPs in each cluster to genes using the SNP2GENE function in FUMA based on positional mapping (with deleterious coding SNPs) (Kircher et al., 2014), eQTL mapping, and chromatin interaction mapping. This three-way mapping strategy is used in

Cluster	# SNPs	Est-T2D	t-T2D	Est-OA	t-OA	Q p-value	I^2
1	411	1.181<br>(0.038)	30.753	0.472<br>(0.028)	16.885	0	0.416
2	72	−0.823<br>(0.093)	8.811	0.576<br>(0.069)	8.367	0	0.880

Table S15: The estimation results using SNPs in each cluster detected by mclust for the causal relationship between BFP and T2D-OA. We report the number of SNPs in the cluster (*#SNPs*), the cluster-specific IVW estimate (*Est*), the standard error (in the parentheses under the estimates) and associated t-statistic (t), and statistics measuring the heterogeneity within each cluster (p-value of the Q statistic and the I^2 statistic).

Cluster	# SNPs	Est-T2D	t-T2D	Est-OA	t-OA	Q p-value	I^2
1	249	1.798<br>(0.047)	37.874	0.606<br>(0.035)	17.370	0.000	0.269
2	238	−0.375<br>(0.053)	7.069	0.363<br>(0.039)	9.412	0	0.811

Table S16: The estimation results using SNPs in each cluster detected by NAvMix with zero initial noise proportion for the causal relationship between BFP and T2D-OA. We report the number of SNPs in the cluster (*#SNPs*), the cluster-specific IVW estimate (*Est*), the standard error (in the parentheses under the estimates) and associated t-statistic (t), and statistics measuring the heterogeneity within each cluster (p-value of the Q statistic and the I^2 statistic).

the applied examples in the original paper introducing FUMA (Watanabe et al., 2017). The uploaded SNPs are also set to be the pre-defined lead SNPs. All default settings are applied, with the exception that we set the reference panel population as "UKB release2b 10k European". For eQTL mapping, following the practice in Grant et al. (2022), we select tissue types from the following data sources: eQTL catalogue, PsychENCODE, van der Wijst et al. scRNA eQTLs, DICE, eQTLGen, Blood eQTLs, MuTHER, xQTLServer, CcomminMind Consortium, BRAINEAC and GTEx v8 (analysts: Aguet François 1 Brown Andrew A. 2 3 4 Castel Stephane E. 5 6 Davis Joe R. 7 8 He Yuan 9 Jo Brian 10 Mohammadi Pejman 5 6 Park YoSon 11 Parsana Princy 12 Segrè Ayellet V. 1 Strober Benjamin J. 9 Zappala Zachary 7 8 et al., 2017; Consortium et al., 2015; Fromer et al., 2016; Grundberg et al., 2012; Ng et al., 2017; Ramasamy et al., 2014; Schmiedel et al., 2018; Van Der Wijst et al., 2018; Võsa et al., 2018; Wang et al., 2018). For chromatin interaction mapping, we select all available Hi-C datasets.

The gene-set enrichment analysis is conducted using the GENE2FUNC function in FUMA. For the mapped genes corresponding to each cluster, we perform the hypergeometric test to check if the mapped genes are over-represented in a pre-defined gene set. Multi-testing correction with the Benjamini-Hochberg procedure is applied, with the adjusted p-value ≤ 0.05 as threshold (Watanabe et al., 2017). The pre-defined gene sets for canonical pathways and gene ontology processes are obtained from MsigDB and WikiPathways (Kutmon et al., 2016; Liberzon et al., 2011). Gene sets for phenotypes are from GWAS

catalog (MacArthur et al., 2017).

To test how the variant clusters are associated with oxidative stress, we create a list of 11 OS biomarkers from various categories. First, as endogenous antioxidants are highly responsive to OS (Tejchman et al., 2021), we use 4 enzyme antioxidants (GST, CAT, SOD, GPX) as OS injury biomarkers, which have been utilized in previous MR studies (Lu et al., 2022; Yifu, 2023). One of the mechanisms through which obesity induces systemic OS is chronic inflammation (Manna and Jain, 2015; Vincent and Taylor, 2006). We thus incorporate three traits known to mediate the pathway from inflammation to OS (CRP, IL-6, TNF-α) (Vincent and Taylor, 2006) as another set of OS biomarkers. Biochemical research has shown that the production of some cytokines, including IL-1β, IL-12 and IL-8, are enhanced under elevated OS levels (Crapo, 2003; Ito et al., 2004). Therefore, we also include these three cytokines in the analysis. Finally, we incorporate GDF-15, which is a biomarker for both inflammation and OS (Wallentin et al., 2014).

The full form of the abbreviations of the biomarkers are as follows: glutathione transferase (GST), catalase(CAT), superoxide dismutase(SOD), glutathione peroxidase (GPX), C-reactive protein (CRP), Interleukin 6 (IL-6), Tumor necrosis factor alpha (TNF-α), Interleukin 1 beta (IL-1β), Interleukin 12 (IL-12), Interleukin 8 (IL-8) and Growth/differentiation factor-15 (GDF-15).

The GWAS summary statistics for the four antioxidants (GST, CAT, SOD, GPX) and CRP are obtained from the GWAS of Sun et al. (2018); for GDF-15, the GWAS of Gudjonsson et al. (2022); for the rest five cytokines, the GWAS of Ahola-Olli et al. (2017) and Kalaoja et al. (2021). Summary statistics for bipolar disorder and major depression disorder are taken from two GWAS studies conducted by the Psychiatric Genomics Consortium (Mullins et al., 2021; Wray, Sullivan, et al., 2017). For MR analyses associated with Cluster 4, the WHR (adjust for BMI) statistics are obtained from a GWAS conducted by the GIANT Consortium (Shungin et al., 2015); for the HDL-C and total

cholesterol data, the GWAS from the Global Lipids Genetics Consortium ("Discovery and refinement of loci associated with lipid levels" 2013); for CAD, the GWAS from the CARDIoGRAMplusC4D Consortium ("A comprehensive 1000 Genomes–based genome-wide association meta-analysis of coronary artery disease" 2015).

	IVW			MR-PRESSO			MR-RAPS		
	Est	SE	Z-score	Est	SE	Z-score	Est	SE	Z-score
GST	0.440	0.216	2.037	0.440	0.207	2.126	0.448	0.221	2.030
CAT	-0.083	0.216	-0.385	-0.083	0.223	-0.373	-0.091	0.227	-0.400
SOD	0.026	0.216	0.120	0.026	0.214	0.121	0.026	0.221	0.120
GPX	0.040	0.216	0.186	0.040	0.223	0.181	0.044	0.226	0.193
CRP	0.545	0.167	3.269	0.545	0.166	3.275	0.552	0.178	3.107
IL-6	0.345	0.141	2.449	0.345	0.148	2.330	0.351	0.145	2.415
TNF-α	0.200	0.215	0.929	0.200	0.215	0.932	0.204	0.220	0.927
IL-1β	0.406	0.220	1.841	0.406	0.232	1.749	0.413	0.235	1.757
IL-12	0.219	0.140	1.561	0.219	0.150	1.455	0.220	0.151	1.461
IL-8	0.345	0.213	1.619	0.345	0.220	1.565	0.354	0.225	1.570
GDF-15	0.264	0.150	1.757	0.264	0.138	1.911	0.268	0.154	1.746
Bipolar	0.232	0.083	2.812	0.127	0.123	1.033	0.226	0.149	1.518
MDD	0.114	0.080	1.425	0.159	0.102	1.558	0.135	0.114	1.183

Table S17: Two-sample MR estimating the effects of BFP on the 11 oxidative stress biomarkers and 2 psychological disorders using variants in Cluster 1 as instruments. Results are given by IVW, MR-PRESSO and MR-RAPS, including the point estimate ("*Est*"), the standard error ("*SE*") and the Z-score (the ratio of the estimate and the standard error).

	IVW			MR-PRESSO			MR-RAPS		
	Est	SE	Z-score	Est	SE	Z-score	Est	SE	Z-score
GST	-0.904	0.491	-1.839	-0.904	0.538	-1.680	-0.979	0.538	-1.819
CAT	-0.601	0.492	-1.223	-0.601	0.513	-1.172	-0.618	0.508	-1.217
SOD	0.878	0.491	1.787	0.878	0.588	1.492	0.778	0.577	1.349
GPX	-0.376	0.492	-0.765	-0.376	0.576	-0.653	-0.173	0.587	-0.294
CRP	0.662	0.337	1.961	0.662	0.308	2.149	0.672	0.345	1.949
IL-6	-0.307	0.311	-0.988	-0.307	0.232	-1.323	-0.310	0.318	-0.975
TNF-α	-0.004	0.484	-0.008	-0.004	0.455	-0.009	-0.004	0.493	-0.008
IL-1β	-0.170	0.495	-0.344	-0.170	0.338	-0.504	-0.171	0.507	-0.338
IL-12	-0.923	0.310	-2.981	-0.923	0.243	-3.796	-0.934	0.318	-2.937
IL-8	-0.259	0.477	-0.543	-0.259	0.441	-0.588	-0.263	0.487	-0.539
GDF-15	-0.899	0.351	-2.564	-0.899	0.478	-1.880	-1.145	0.545	-2.101
Bipolar	0.208	0.189	1.104	0.208	0.207	1.008	0.224	0.209	1.069
MDD	0.092	0.179	0.513	0.092	0.207	0.442	0.146	0.216	0.674
WHRadj	-0.529	0.101	-5.208	-0.634	0.249	-2.550	-0.476	0.281	-1.692
HDL-C	0.926	0.097	9.500	1.280	0.215	5.949	0.932	0.234	3.990
TC	-0.414	0.101	-4.096	-0.192	0.104	-1.853	-0.395	0.139	-2.838
CAD	-0.887	0.416	-2.131	-0.887	0.433	-2.049	-0.896	0.425	-2.108

Table S18: Two-sample MR estimating the effects of BFP on the 11 oxidative stress biomarkers, 2 psychological disorders, WHR (adjusted for BMI), HDL-C, total cholesterol (TC) and CAD using variants in Cluster 4 as instruments. Results are given by IVW, MR-PRESSO and MR-RAPS, including the point estimate ("*Est*"), the standard error ("*SE*") and the Z-score (the ratio of the estimate and the standard error).

References

Ahola-Olli, A. V., Würtz, P., Havulinna, A. S., Aalto, K., Pitkänen, N., Lehtimäki, T., Kähönen, M., Lyytikäinen, L.-P., Raitoharju, E., Seppälä, I., et al. (2017). Genome-wide association study identifies 27 loci influencing concentrations of circulating cytokines and growth factors. *The American Journal of Human Genetics*, *100*(1), 40–50.

analysts: Aguet François 1 Brown Andrew A. 2 3 4 Castel Stephane E. 5 6 Davis Joe R. 7 8 He Yuan 9 Jo Brian 10 Mohammadi Pejman 5 6 Park YoSon 11 Parsana Princy 12 Segrè Ayellet V. 1 Strober Benjamin J. 9 Zappala Zachary 7 8, G. C. L., program management: Addington Anjene 15 Guan Ping 16 Koester Susan 15 Little A. Roger 17 Lockhart Nicole C. 18 Moore Helen M. 16 Rao Abhi 16 Struewing Jeffery P. 19 Volpi Simona 19, N., 16, P. S. L. 3. B. M. E. 3. B. P. A., 137, N. C. F. N. C. R., et al. (2017). Genetic effects on gene expression across human tissues. *Nature*, *550*(7675), 204–213.

Andrews, D. W. K. (1999). Consistent moment selection procedures for generalized method of moments estimation. *Econometrica*, *67*(3), 543–563. https://doi.org/10.1111/1468-0262.00036

Apfel, N., & Liang, X. (2021). Agglomerative hierarchical clustering for selecting valid instrumental variables. *arXiv preprint arXiv:2101.05774*.

Belloni, A., Chen, D., Chernozhukov, V., & Hansen, C. (2012). Sparse models and methods for optimal instruments with an application to eminent domain. *Econometrica*, *80*(6), 2369–2429. https://doi.org/10.3982/ecta9626

Bi, N., Kang, H., & Taylor, J. (2019). Inference after selecting plausibly valid instruments with application to mendelian randomization. *arXiv preprint arXiv:1911.03985*.

Bowden, J., Del Greco M, F., Minelli, C., Zhao, Q., Lawlor, D. A., Sheehan, N. A., Thompson, J., & Davey Smith, G. (2019). Improving the accuracy of two-sample summary-data mendelian randomization: Moving beyond the nome assumption. *International journal of epidemiology*, *48*(3), 728–742.

Bowden, J., & Holmes, M. V. (2019). Meta-analysis and mendelian randomization: A review. *Research Synthesis Methods*, *10*(4), 486–496. https://doi.org/https://doi.org/10.1002/jrsm.1346

Bulik-Sullivan, B., Finucane, H. K., Anttila, V., Gusev, A., Day, F. R., Loh, P.-R., Duncan, L., Perry, J. R., Patterson, N., Robinson, E. B., et al. (2015). An atlas of genetic correlations across human diseases and traits. *Nature genetics*, *47*(11), 1236–1241.

Burgess, S., Smith, G. D., Davies, N. M., Dudbridge, F., Gill, D., Glymour, M. M., Hartwig, F. P., Kutalik, Z., Holmes, M. V., Minelli, C., et al. (2019). Guidelines for performing mendelian randomization investigations: Update for summer 2023. *Wellcome open research*, *4*.

A comprehensive 1000 genomes–based genome-wide association meta-analysis of coronary artery disease. (2015). *Nature genetics*, *47*(10), 1121–1130.

Consortium, G., Ardlie, K. G., Deluca, D. S., Segrè, A. V., Sullivan, T. J., Young, T. R., Gelfand, E. T., Trowbridge, C. A., Maller, J. B., Tukiainen, T., et al. (2015). The genotype-tissue expression (gtex) pilot analysis: Multitissue gene regulation in humans. *Science*, *348*(6235), 648–660.

Crapo, J. (2003). Oxidative stress as an initiator of cytokine release and cell damage. *European Respiratory Journal*, *22*(44 suppl), 4s–6s.

Discovery and refinement of loci associated with lipid levels. (2013). *Nature genetics*, *45*(11), 1274–1283.

Foley, C. N., Mason, A. M., Kirk, P. D., & Burgess, S. (2021). Mr-clust: Clustering of genetic variants in mendelian randomization with similar causal estimates. *Bioinformatics*, *37*(4), 531–541.

Fromer, M., Roussos, P., Sieberts, S. K., Johnson, J. S., Kavanagh, D. H., Perumal, T. M., Ruderfer, D. M., Oh, E. C., Topol, A., Shah, H. R., et al. (2016). Gene expression elucidates functional impact of polygenic risk for schizophrenia. *Nature neuroscience*, *19*(11), 1442–1453.

Grant, A. J., Gill, D., Kirk, P. D., & Burgess, S. (2022). Noise-augmented directional clustering of genetic association data identifies distinct mechanisms underlying obesity. *PLoS Genetics*, *18*(1), e1009975.

Greco M, F. D., Minelli, C., Sheehan, N. A., & Thompson, J. R. (2015). Detecting pleiotropy in mendelian randomisation studies with summary data and a continuous outcome. *Statistics in medicine*, *34*(21), 2926–2940.

Grundberg, E., Small, K. S., Hedman, Å. K., Nica, A. C., Buil, A., Keildson, S., Bell, J. T., Yang, T.-P., Meduri, E., Barrett, A., et al. (2012). Mapping cis-and trans-regulatory effects across multiple tissues in twins. *Nature genetics*, *44*(10), 1084–1089.

Gudjonsson, A., Gudmundsdottir, V., Axelsson, G. T., Gudmundsson, E. F., Jonsson, B. G., Launer, L. J., Lamb, J. R., Jennings, L. L., Aspelund, T., Emilsson, V., et al. (2022). A genome-wide association study of serum proteins reveals shared loci with common diseases. *Nature communications*, *13*(1), 480.

Guo, Z. (2023). Causal inference with invalid instruments: Post-selection problems and a solution using searching and sampling. *Journal of the Royal Statistical Society Series B: Statistical Methodology*, *85*(3), 959–985.

Guo, Z., Kang, H., Tony Cai, T., & Small, D. S. (2018). Confidence intervals for causal effects with invalid instruments by using two-stage hard thresholding with voting. *Journal of the Royal Statistical Society Series B: Statistical Methodology*, *80*(4), 793–815.

Ito, K., Hanazawa, T., Tomita, K., Barnes, P., & Adcock, I. (2004). Oxidative stress reduces histone deacetylase 2 activity and enhances il-8 gene expression: Role of tyrosine nitration. *Biochemical and biophysical research communications*, *315*(1), 240–245.

Kalaoja, M., Corbin, L. J., Tan, V. Y., Ahola-Olli, A. V., Havulinna, A. S., Santalahti, K., Pitkänen, N., Lehtimäki, T., Lyytikäinen, L.-P., Raitoharju, E., et al. (2021). The role of inflammatory cytokines as intermediates in the pathway from increased adiposity to disease. *Obesity*, *29*(2), 428–437.

Kircher, M., Witten, D. M., Jain, P., O'roak, B. J., Cooper, G. M., & Shendure, J. (2014). A general framework for estimating the relative pathogenicity of human genetic variants. *Nature genetics*, *46*(3), 310–315.

Kutmon, M., Riutta, A., Nunes, N., Hanspers, K., Willighagen, E. L., Bohler, A., Mélius, J., Waagmeester, A., Sinha, S. R., Miller, R., et al. (2016). Wikipathways: Capturing the full diversity of pathway knowledge. *Nucleic acids research*, *44*(D1), D488–D494.

Leeb, H., & Pötscher, B. M. (2005). Model selection and inference: Facts and fiction. *Econometric Theory*, *21*(1), 21–59.

Liberzon, A., Subramanian, A., Pinchback, R., Thorvaldsdóttir, H., Tamayo, P., & Mesirov, J. P. (2011). Molecular signatures database (msigdb) 3.0. *Bioinformatics*, *27*(12), 1739–1740.

Lu, Z., Pu, C., Zhang, Y., Sun, Y., Liao, Y., Kang, Z., Feng, X., & Yue, W. (2022). Oxidative stress and psychiatric disorders: Evidence from the bidirectional mendelian randomization study. *Antioxidants*, *11*(7), 1386.

MacArthur, J., Bowler, E., Cerezo, M., Gil, L., Hall, P., Hastings, E., Junkins, H., McMahon, A., Milano, A., Morales, J., et al. (2017). The new nhgri-ebi catalog of published genome-wide association studies (gwas catalog). *Nucleic acids research*, *45*(D1), D896–D901.

Manna, P., & Jain, S. K. (2015). Obesity, oxidative stress, adipose tissue dysfunction, and the associated health risks: Causes and therapeutic strategies. *Metabolic syndrome and related disorders*, *13*(10), 423–444.

Mullins, N., Forstner, A. J., O'Connell, K. S., Coombes, B., Coleman, J. R., Qiao, Z., Als, T. D., Bigdeli, T. B., Børte, S., Bryois, J., et al. (2021). Genome-wide association study of more than 40,000 bipolar disorder cases provides new insights into the underlying biology. *Nature genetics*, *53*(6), 817–829.

Ng, B., White, C. C., Klein, H.-U., Sieberts, S. K., McCabe, C., Patrick, E., Xu, J., Yu, L., Gaiteri, C., Bennett, D. A., et al. (2017). An xqtl map integrates the genetic architecture of the human brain's transcriptome and epigenome. *Nature neuroscience*, *20*(10), 1418–1426.

Patel, A., Ditraglia, F. J., Zuber, V., & Burgess, S. (2021). Selection of invalid instruments can improve estimation in mendelian randomization. *Arxiv. http://arxiv.org/abs/2107.01513*.

Ramasamy, A., Trabzuni, D., Guelfi, S., Varghese, V., Smith, C., Walker, R., De, T., Robert, U. B. E. C. H. J. R. M. T. D. G. S. W. M. E. R. A. F. P. 1. S. C. W., Coin, L., et al. (2014). Genetic variability in the regulation of gene expression in ten regions of the human brain. *Nature neuroscience*, *17*(10), 1418–1428.

Rand, W. M. (1971). Objective criteria for the evaluation of clustering methods. *Journal of the American Statistical association*, *66*(336), 846–850.

Schmiedel, B. J., Singh, D., Madrigal, A., Valdovino-Gonzalez, A. G., White, B. M., Zapardiel-Gonzalo, J., Ha, B., Altay, G., Greenbaum, J. A., McVicker, G., et al. (2018). Impact of genetic polymorphisms on human immune cell gene expression. *Cell*, *175*(6), 1701–1715.

Shungin, D., Winkler, T. W., Croteau-Chonka, D. C., Ferreira, T., Locke, A. E., Mägi, R., Strawbridge, R. J., Pers, T. H., Fischer, K., Justice, A. E., et al. (2015). New genetic loci link adipose and insulin biology to body fat distribution. *Nature*, *518*(7538), 187–196.

Staiger, D., & Stock, J. H. (1997). Instrumental variables regression with weak instruments. *Econometrica*, *65*(3), 557–586.

Sun, B. B., Maranville, J. C., Peters, J. E., Stacey, D., Staley, J. R., Blackshaw, J., Burgess, S., Jiang, T., Paige, E., Surendran, P., et al. (2018). Genomic atlas of the human plasma proteome. *Nature*, *558*(7708), 73–79.

Tejchman, K., Kotfis, K., & Sieńko, J. (2021). Biomarkers and mechanisms of oxidative stress—last 20 years of research with an emphasis on kidney damage and renal transplantation. *International journal of molecular sciences*, *22*(15), 8010.

Van Der Wijst, M. G., Brugge, H., De Vries, D. H., Deelen, P., Swertz, M. A., Study, L. C., Consortium, B., & Franke, L. (2018). Single-cell rna sequencing identifies celltype-specific cis-eqtls and co-expression qtls. *Nature genetics*, *50*(4), 493–497.

Vincent, H. K., & Taylor, A. G. (2006). Biomarkers and potential mechanisms of obesity-induced oxidant stress in humans. *International journal of obesity*, *30*(3), 400–418.

Võsa, U., Claringbould, A., Westra, H.-J., Bonder, M. J., Deelen, P., Zeng, B., Kirsten, H., Saha, A., Kreuzhuber, R., Kasela, S., et al. (2018). Unraveling the polygenic architecture of complex traits using blood eqtl metaanalysis. *BioRxiv*, 447367.

Wallentin, L., Hijazi, Z., Andersson, U., Alexander, J. H., De Caterina, R., Hanna, M., Horowitz, J. D., Hylek, E. M., Lopes, R. D., Åsberg, S., et al. (2014). Growth differentiation factor 15, a marker of oxidative stress and inflammation, for risk assessment in patients with atrial fibrillation: Insights from the apixaban for reduction in stroke and other thromboembolic events in atrial fibrillation (aristotle) trial. *Circulation*, *130*(21), 1847–1858.

Wang, D., Liu, S., Warrell, J., Won, H., Shi, X., Navarro, F. C., Clarke, D., Gu, M., Emani, P., Yang, Y. T., et al. (2018). Comprehensive functional genomic resource and integrative model for the human brain. *Science*, *362*(6420), eaat8464.

Wang, J., Zhao, Q., Bowden, J., Hemani, G., Davey Smith, G., Small, D. S., & Zhang, N. R. (2021). Causal inference for heritable phenotypic risk factors using heterogeneous genetic instruments. *PLoS genetics*, *17*(6), e1009575.

Wang, S., & Kang, H. (2022). Weak-instrument robust tests in two-sample summary-data mendelian randomization. *Biometrics*, *78*(4), 1699–1713.

Watanabe, K., Taskesen, E., Van Bochoven, A., & Posthuma, D. (2017). Functional mapping and annotation of genetic associations with fuma. *Nature communications*, *8*(1), 1826.

Windmeijer, F., Liang, X., Hartwig, F. P., & Bowden, J. (2021). The confidence interval method for selecting valid instrumental variables. *Journal of the Royal Statistical Society: Series B (Statistical Methodology)*, *83*(4), 752–776.

Wray, N. R., Sullivan, P. F., et al. (2017). Genome-wide association analyses identify 44 risk variants and refine the genetic architecture of major depression. biorxiv. *URL https://www. biorxiv. org/content/early/2017/07/24/167577.*

Yifu, P. (2023). Evidence for causal effects of polycystic ovary syndrome on oxidative stress: A two-sample mendelian randomisation study. *BMC Medical Genomics*, *16*(1), 141.

Zhao, Q., Wang, J., Hemani, G., Bowden, J., & Small, D. S. (2020). Statistical inference in two-sample summary-data mendelian randomization using robust adjusted profile score. *The Annals of Statistics*, *48*(3), 1742–1769.
